# Taxogenomics Resolves Conflict in the Genus *Rhodobacter*: A Two and Half Decades Pending Thought to Reclassify the Genus *Rhodobacter*

**DOI:** 10.3389/fmicb.2019.02480

**Published:** 2019-10-31

**Authors:** G. Suresh, Tushar D. Lodha, B. Indu, Ch. Sasikala, Ch. V. Ramana

**Affiliations:** ^1^Department of Plant Sciences, School of Life Sciences, University of Hyderabad, Hyderabad, India; ^2^Bacterial Discovery Laboratory, Centre for Environment, Institute of Science and Technology, Jawaharlal Nehru Technological University Hyderabad, Hyderabad, India

**Keywords:** *Rhodobacter*, taxogenomics, *Rhodobacter sensu stricto*, gen. nov., *Rhodobacter* reclassification, phylogenomics, proposal of 3 new phototrophic genera, photosynthetic gene cluster

## Abstract

The genus *Rhodobacter* is taxonomically well studied, and some members are model organisms. However, this genus is comprised of a heterogeneous group of members. 16S rRNA gene-based phylogeny of the genus *Rhodobacter* indicates a motley assemblage of anoxygenic phototrophic bacteria (genus *Rhodobacter*) with interspersing members of other genera (chemotrophs) making the genus polyphyletic. Taxogenomics was performed to resolve the taxonomic conflicts of the genus *Rhodobacter* using twelve type strains. The phylogenomic analysis showed that *Rhodobacter* spp. can be grouped into four monophyletic clusters with interspersing chemotrophs. Genomic indices (ANI and *d*DDH) confirmed that all the current species are well defined, except *Rhodobacter megalophilus*. The average amino acid identity values between the monophyletic clusters of *Rhodobacter* members, as well as with the chemotrophic genera, are less than 80% whereas the percentage of conserved proteins values were below 70%, which has been observed among several genera related to *Rhodobacter*. The pan-genome analysis has shown that there are only 1239 core genes shared between the 12 species of the genus *Rhodobacter*. The polyphasic taxonomic analysis supports the phylogenomic and genomic studies in distinguishing the four *Rhodobacter* clusters. Each cluster is comprised of one to seven species according to the current *Rhodobacter* taxonomy. Therefore, to address this taxonomic discrepancy we propose to reclassify the members of the genus *Rhodobacter* into three new genera, *Luteovulum* gen. nov., *Phaeovulum* gen. nov. and *Fuscovulum* gen. nov., and provide an emended description of the genus *Rhodobacter sensu stricto*. Also, we propose reclassification of *Rhodobacter megalophilus* as a sub-species of *Rhodobacter sphaeroides*.

## Introduction

The genus *Rhodobacter* (*Rba*.) was proposed by Imhoff et al. (1984) to accommodate those species of *Rhodopseudomonas* (*Rhodopseudomonas capsulata* [formerly *Rhodonostoc capsulatum* (Molisch, 1907)], *Rhodopseudomonas spheroides*, *Rhodopseudomonas sulfidophila* and *Rhodopseudomonas adriatica*) with vesicular intracytoplasmic membrane (ICM) architecture as *Rba. capsulatus* (type species of the genus), *Rba. sphaeroides*, *Rba. adriaticus* and *Rba. sulfidophilus.* Subsequently, based on 16S rRNA gene sequence comparisons, requirement of NaCl for optimal growth, final oxidation product of sulfide, polar lipid composition and sulfide tolerance, *Rba. sulfidophilus*, *Rba*. *euryhalinus*, and *Rba. adriaticus* were reclassified as *Rhodovulum sulfidophilum*, *Rhodovulum euryhulinum*, and *Rhodovulum adriaticum* (Hiraishi and Ueda, 1994). *Rhodopseudomonas blastica* was also reclassified into the genus *Rhodobacter*, although it should be emphasized that *Rba. blasticus* is now the only member of the genus with a lamellar ICM architecture (Hiraishi and Ueda, 1994).

*Rhodobacter* is the type genus of the family ‘Rhodobacteraceae’ which presently contains more than 150 validly named genera^1^. However, it must be noted that the name ‘Rhodobacteraceae’ is illegitimate^2^ as the relationship between this suprageneric grouping and the family *Hyphomonadaceae* (Lee et al., 2005) has not yet been resolved. Lee et al. (2005) grouped members of the family ‘Rhodobacteraceae’ into five-well defined groups, based on phylogenetic analysis. These are the *Rhodobacter*, *Rhodovulum*, *Amaricoccus*, *Roseobacter*, and *Paracoccus* groups. The *Rhodobacter* group consists of 17 genera: *Albirhodobacter, Cereibacter*, *Defluviimonas, Falsirhodobacter, Gemmobacter, Haematobacter, Paenirhodobacter, Pararhodobacter, Plastorhodobacter, Pseudorhodobacter, Rhodobaca, Rhodobacter, Roseicitreum, Roseibaca, Roseinatronobacter, Sinorhodobacter*, and *Thioclava*.

Members of the genus *Rhodobacter* perform anoxygenic photosynthesis, fix dinitrogen and play a key role in bio-geochemical cycles. At present, the genus *Rhodobacter* comprises 16 validly named species: *Rba. aestuarii*, *Rba. azollae, Rba. alkalitolerans, Rba. azotoformans*, *Rba*. *blasticus*, *Rba. capsulatus*, *Rba*. *johrii*, *Rba. lacus, Rba. maris*, *Rba. megalophilus*, *Rba. ovatus*, *Rba. sediminis, Rba*. *sphaeroides*, *Rba. veldkampii*, *Rba. Vinaykumarii*, and *Rba. viridis*. Species of the genus *Rhodobacter* have ovoid to rod-shaped-cells, are Gram-stain negative, facultative photoheterotrophic and have vesicular/lamellar ICM architecture, bacteriochlorophyll-*a*, carotenoids of the spheroidene series and have Q10 as their major quinone. Some of the members are capable of growing photolithoautotrophically in the presence of sulfide/H_2_ as an electron donor (Imhoff, 2005; Girija et al., 2010; Suresh et al., 2017; Gandham et al., 2018).

Based on 16S rRNA gene sequence phylogenetic analysis we previously demonstrated the heterogeneity of the genus *Rhodobacter*, due to a large diversity of interspersing chemotrophs dividing the members into five monophyletic clusters (Suresh et al., 2017). Apart from being polyphyletic, the members of the genus *Rhodobacter* also have phenotypic differences which mainly include: heterogeneity in polar lipids (presence and absence glycolipids/diphosphatidylglycerol [DPG]), ICM architecture (vesicular/lamellar), sulfur metabolism and cell division (budding/binary fission). The phenotypic diversity among the taxa of the genus *Rhodobacter* was critically commented upon 21/2 decades back (Hiraishi and Ueda, 1994). However, due to a lack of strong evidence, reclassification of this genus has not yet been done. A taxogenomics (phylogenomics) helped in the reclassification of *Mycobacterium* (Gupta et al., 2018), *Phaeobacter* (Breider et al., 2014), Gammaproteobacterial methanotrophs (Orata et al., 2018), *Geobacillus* (Aliyu et al., 2016), *Burkholderia* (Lopes-Santos et al., 2017), *Populibacter* (Li et al., 2017), and *Roseobacter* group (Wirth and Whitman, 2018). In the present communication, based on phylogenetic, pan-genomic, phylogenomic/taxogenomic analysis supported by phenotypic and chemotaxonomic differences, we propose the reclassification of the genus *Rhodobacter* into three new genera and emended description of genus *Rhodobacter sensu stricto*.

## Materials and Methods

### Organisms and Growth Conditions

All the 16 current type species of the genus *Rhodobacter* were cultured and maintained on the medium described by Lakshmi et al. (2011) in completely filled screw cap test tubes (10 × 100 mm) under photoheterotrophic conditions using pyruvate as a carbon source. Pure cultures were maintained on agar slants or as lyophilized cultures were preserved at 4°C.

### Organisms and Genome Sequences

Type strains of twelve species of the genus *Rhodobacter* and type species of different genera of the *Rhodobacter* group in the family ‘Rhodobacteraceae’ (Pujalte et al., 2014), whose genomes were available along with representatives from each of the remaining four groups (Lee et al., 2005) were considered in this study. *Escherichia coli* ATCC 11775^T^ and *Pseudomonas aeruginosa* DSM 50071^T^ genomes were included as out groups. The genome sequences of type strains of *Rba. blasticus*, *Rba. veldkampii* and *Cereibacter changlensis* were shared by Prof. Meyer Terrance, prior to their release to the NCBI database. All the other genome sequences used in the present study were downloaded from the NCBI database (Supplementary Table S1).

### Phylogenetic Analysis

Phylogenetic analysis was performed with MEGA7 software (Kumar et al., 2016). For 16S rRNA gene-based phylogenetic analysis, sequences of all *Rhodobacter* spp. and related members were extracted from the NCBI database. From all 32 genomes selected in this study including the two outgroups, ninety-two core genes were retrieved using the Up-to-date Bacterial Core Gene (UBCG) tool (Na et al., 2018). A concatenated sequence was used to construct the phylogenetic tree. For both trees (16S rRNA gene and UBCG tree) distances were calculated by using the Kimura2-parameter (Kimura, 1980) in a pairwise deletion procedure and Poisson model (Zuckerkandl and Pauling, 1965) for the protein sequences of PufX, PufL,M and photosynthetic gene cluster (PGC) based phylogenetic trees. Neighbor-Joining (NJ), Maximum Likelihood (ML) and Minimum Evolution (ME) methods in the MEGA7 software were used to construct phylogenetic trees. Bootstrap analysis was carried out with 1000 resampling.

The phylogenomic tree was also deduced from the genome translated amino acids using the automated pipeline of the Patric online server^3^. In brief, the Patric server begins with amino acid sequence files for each genome. Homologous proteins were identified in two rounds. BLAST was used in first round in which the genome of each distinct species is searched against other genomes and an MCL (Markov Cluster) algorithm was used to cluster the top scoring hits, which are initial seed sets for the homology groups. Alignment of seed sets was carried out by MUSCLE, and hmmbuild was used to build Hidden Markov Models (HMMs). The phylogenomic tree was generated with FastTree and RAxML from the concatenated alignment. Instead of bootstraps, trees are built from random samples of 50% of the homology groups used for the main tree, in a process referred to as gene-wise jackknifing. Hundred of these 50% gene-wise jackknife trees are made using FastTree/RAxML, and the support values shown indicate the number of times a particular branch was observed in the support trees.

### Analysis of Pan-Genome and Genomic Indices

The pan-genome analysis of genomes of type strains of 12 species of the genus *Rhodobacter* was carried out using the BPGA pipeline (Chaudhari et al., 2015), with default parameters to check the inter species variation and core genome. The pan-genome analysis was also carried out for proposed genera. In order to ensure the correct assignation at the species level of each analyzed genome, the ANI and the *d*DDH were calculated between the genomes. The OrthoANIu values were calculated using the OrthoANI tool (Yoon et al., 2017). The *d*DDH values were calculated with the GGDC software and values obtained with the formula 2 were considered (Auch et al., 2012). Average amino acid identity (AAI) and percentage of conserved protein (POCP) was calculated among the selected genomes as these are considered as important for genus classification. The AAI was calculated using the AAI calculator developed by Kostas lab (Rodriguez and Konstantinidis, 2014). The POCP was determined as described by Qin et al. (2014).

### Polar Lipid Analysis

Polar lipids were extracted from 1g freeze-dried cells with methanol/chloroform/saline (2:1:0.8, by vol.) as described by Kates (1986). Lipids were separated using silica gel TLC (Kieselgel 60 F_254_; Merck) by two-dimensional chromatography using chloroform/methanol/water (65:25:4 by vol.) in the first dimension and chloroform/methanol/acetic acid/water (80:12:15:4 by vol.) in the second dimension (Tindall, 1990a, b). The total lipid profile was visualized by spraying with 5% ethanolic molybdophosphoric acid and was further characterized by spraying with ninhydrin (specific for amino groups), molybdenum blue (specific for phosphates), Dragendorff (quaternary nitrogen), α-naphthol (specific for sugars) (Oren et al., 1996; Kates, 1972).

## Results

### Phylogenetic Analysis Using 16S rRNA Gene Sequences

**FIGURE 1:**
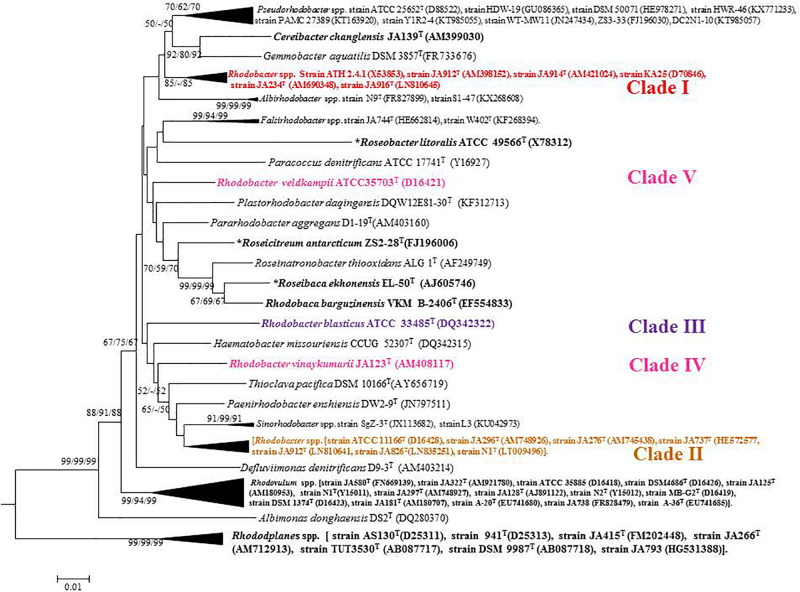
Phylogenetic tree based on 16S rRNA gene sequences showing the phylogenetic relationships of the genus *Rhodobacter* of the family ‘Rhodobacteraceae.’ The tree was computed with MEGA 7 software and rooted by using *Escherichia coli* and *Pseudomonas aeruginosa* as out-group. The GenBank accession numbers for 16S rRNA gene sequences are shown in parentheses. Bootstrap percentages refer to NJ/ML/ME analysis. Bar, 0.01 nucleotide substitution per position. Phototrophic bacteria are indicated by bold letters and aerobic anoxygenic phototrophic bacteria with bold letters and star.

Based on 16S rRNA gene sequence phylogenetic analysis, the sixteen species of the genus *Rhodobacter* form five distinct clades (Figure 1) with interspersing chemotrophs (members of other described genera in the family ‘Rhodobacteraceae’). Clade I, the *Rba*. *sphaeroides* clade, contain *Rba*. *sphaeroides*, *Rba*. *johrii*, *Rba. megalophilus*, *Rba. azotoformans*, *Rba. ovatus* and *Rba. alkalitolerans.* Clade II contain the *Rba. capsulatus* clade, which includes *Rba. capsulatus, Rba. viridis, Rba. azollae, Rba. sediminis*, *Rba. aestuarii*, *Rba. maris*, and *Rba. lacus.* Clade III, the *Rba*. *blasticus*, clade contains only a single species, *Rba*. *blasticus*. Clade IV is the *Rba. veldkampii* clade containing only *Rba. veldkampii* while clade V is represented by *Rba. vinaykumarii.* The 16S rRNA gene pairwise sequence similarities among *Rhodobacter* species were calculated using LALIGN tool^4^ and the results showed that some of the *Rhodobacter* species (between clade I and clade II) have 94.0% sequence similarity (Supplementary Table S2), which is less than the recommended value for genus delineation (Rosselló-Móra and Amann, 2015).

### Genomic Features of *Rhodobacter* spp.

**FIGURE 2:**
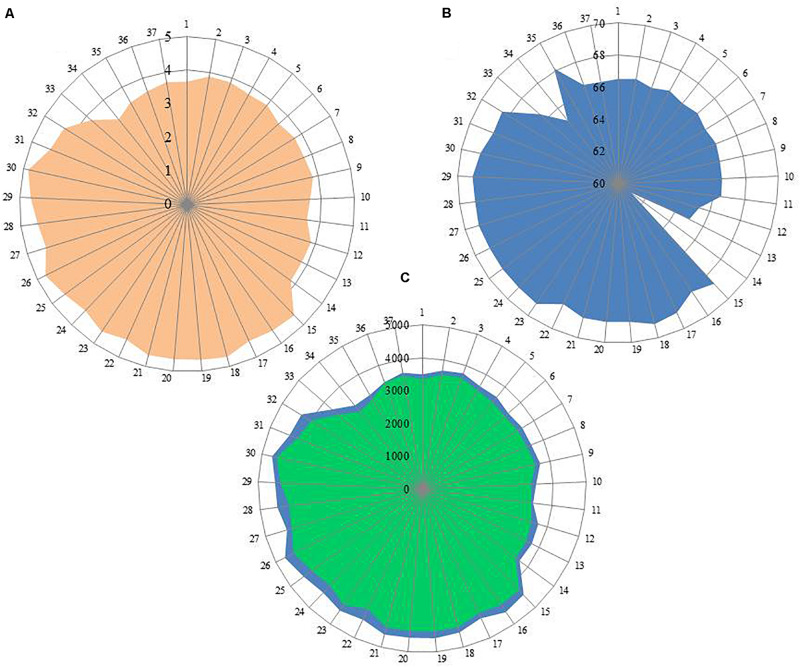
Genomic information of genus *Rhodobacter* members. **(A)** Genome size (Mb); **(B)** G+C content calculated from Genome in mol%; **(C)** No of genes in blue color and no of proteins in green color. Each spoke in the circle represent one strain and the numbering same as in Supplementary Table S3.

**FIGURE 3:**
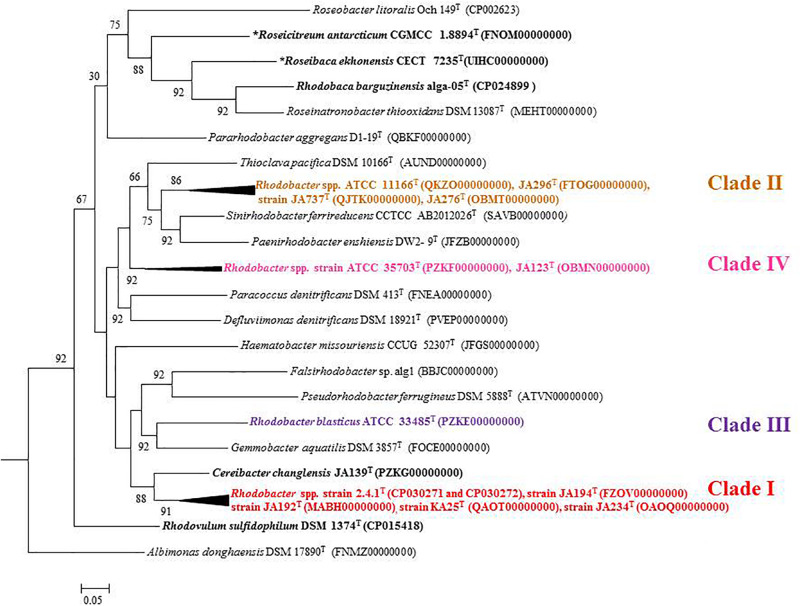
Phylogenetic tree constructed using the 92 bacterial core gene sequences. The tree was constructed using MEGA 7 software. Bootstrap percentages refer to NJ/ML/ME analysis. Phototrophic bacteria are indicated by bold letters and aerobic anoxygenic phototrophic bacteria with bold letters and star.

Out of 16 type strains of *Rhodobacter*, genome sequences of 12 type strains are available. Genome sequences of *Rba. alkalitolerans* (Gandham et al., 2018), *Rba. azollae, Rba. lacus* (Suresh et al., 2017) and *Rba. sediminis* (Subhash and Lee, 2016) are not available. A genomic summary of the twelve species (and some related strains) is presented in Figure 2 and Supplementary Table S3. Clade I members typically have large genomes (4.3–4.7 Mb) and high G+C content (68.2–69.1 mol%). An exception in Clade I is *Rba. ovatus*, which has an 3.8 Mb genome and genomic GC content of 66.5 mol%. Clade II members have 3.6–3.9 Mb genomes and genomic GC content of 61.0–66.6 mol%. Clade III members have a genome size of 3.6–3.7 Mb, 66.4–66.5 GC mol%, while clade IV and V have 3.3, 3.5 Mb and 65.0, 68.2 GC mol%, respectively. Phylogenetic analysis based on 92 bacterial core genes (UBCG) also showed (Figure 3) that members of *Rhodobacter* are not monophyletic, as observed in the 16S rRNA gene-based phylogenetic tree (Figure 1), with the exception of *Rba. veldkampii* and *Rba. vinaykumarii* forming one cluster unlike in the 16S rRNA gene-based phylogenetic tree.

### Analysis of Core and Pan-Genome of the Genus *Rhodobacter*

Bacterial pan-genome analysis was carried out between the type strains of the genus *Rhodobacter*. In the 12 analyzed species of the genus *Rhodobacter*, 1239 genes were identified as core genes. The species of the *Rba. sphaeroides* clade have 2294 genes as a core genome with 6256 genes as accessory (Supplementary Figure S1), whereas species of the *Rba. capsulatus* clade share 2225 genes as a core genome with 2588 genes as the accessory genome (Supplementary Figure S2). The distribution of core genome, accessory genome and unique genes is given in Supplementary Table S4.

### Digital DNA–DNA Hybridization and Average Nucleotide Identity (ANI)

*d*DDH values were calculated between 12 species of genus *Rhodobacter* and other closely related genera. *d*DDH values ranged from 18.6 to 59.0% between type strains of the genus *Rhodobacter* (Supplementary Table S5) except between the pair of *Rba. sphaeroides* 2.4.1^T^ and *Rba*. *megalophilus* JA194^T^ (81.6%). The results of *d*DDH also matched with the data obtained using the Type (Strain) Genome Server (TYGS) (Meier-Kolthoff and Göker, 2019). Orthologous Average Nucleotide Identity (OrthoANI) varied between 68.25 and 94.93% between type strains of the genus *Rhodobacter.* The only exception was again the pair, *Rba. sphaeroides* 2.4.1^T^ and *Rba*. *megalophilus* JA194^T^, where it was 97.96% (Supplementary Table S5).

### POCP and AAI Values for Genera Delineation

A POCPs threshold below 50% was proposed to determine if a species belongs to the two different genera (Qin et al., 2014). The POCP values within the genus *Rhodobacter* ranged from 54.2 to 88.2%. The lowest POCP value was between *Rba*. *megalophilus* JA194^T^ and *Rba. aestuarii* JA296^T^ and highest POCP value was between *Rba*. *megalophilus* JA194^T^ and *Rba. sphaeroides* 2.4.1^T^. The POCP values among the clade I species ranged from 66.5 to 88.2%, whereas for clade II values were between 72.1 to 79.9%, and was 71.1% between *Rba. veldkampii* and *Rba. vinaykumarii*. The POCP value between *Rba. capsulatus* ATCC 11166^T^ and *Rba. sphaeroides* 2.4.1^T^ was 59%. POCP values among *Rhodobacter* clades and related genera are above 50% (Supplementary Table S6).

The AAI among the different clades of *Rhodobacter* along with chemotrophs were below 80% (Supplementary Table S6). The AAI values within the genus *Rhodobacter* ranged from 63.04 to 98.56%. The lowest AAI value was between *Rba*. *megalophilus* JA194^T^ and *Rba. aestuarii* JA296^T^ and highest AAI value was between *Rba*. *megalophilus* JA194^T^ and *Rba. sphaeroides* 2.4.1^T^. The AAI values in clade I range from 98.56 to 80.37%, whereas clade II values range from 77.86% to 89.21%, and 72.85% between *Rba. veldkampii* and *Rba. vinaykumarii*. The AAI value between the *Rba. capsulatus* ATCC 11166^T^ and *Rba. sphaeroides* 2.4.1^T^ was 64.36%. AAI values between the type species of all the genera under study were below the recommended cut-off value of 80% (Luo et al., 2014; Supplementary Table S6). The average AAI value between the members of clade I and clade II was 64.1%, between clade I and clade III was 66.64%, between clade I and clade IV was 67.18%, between clade II and clade III was 63.72%, between clade II and IV was 68.53% and between the clade III and IV was 65.14%. These values are well below the recommended cut-off of 80% used for the genus delineation (Luo et al., 2014).

### Analysis of Photosynthesis Related Genes of *Rhodobacter* spp.

#### PufLM Protein Phylogenetic Analysis

PufLM proteins are essential components of bacteria which perform photosynthesis with the help of photosystem II type of photosynthetic apparatus, and the phylogeny of both anaerobic and aerobic photosynthetic bacteria can be studied by PufLM protein analysis (Imhoff et al., 2018). Full length amino acid sequences of PufLM of 12 *Rhodobacter* type strains were extracted from their genomes and a phylogenetic tree was (Supplementary Figure S3) constructed using *R. sulfidophilum* as the out-group. All the *Rhodobacter* members were recovered in four different clades, similar to the 16S rRNA gene based phylogenetic tree although *Rba. veldkampii* and *Rba. vinaykumarii* formed a single cluster, as in the phylogenomic tree (Figure 3).

#### Photosynthetic Reaction Center Protein (PufX)

Crouch and Jones (2012) showed that some of the current members of the genus *Rhodobacter* (*Rba. sphaeroides*, *Rba. azotoformans*, *Rba. blasticus*) and *C. changlensis* (formerly *Rba. changlensis*) form RC-LH1 (photosynthesis reaction center-light-harvesting 1) complex dimers instead of monomers, as in *Rba. capsulatus*, *Rba. veldkampii* and *Rba*. *vinaykumarii*. The dimer formation is facilitated by the PufX protein, by interacting with two monomers of RC–LH1 complex, without disturbing their structure (Crouch and Jones, 2012). Deletion of the *pufX* gene in *Rba. sphaeroides* resulted in the formation of only RC-LH1 monomers and loss of the ability to grow phototrophically. However, in contrast, *Rba. veldkampii* in spite of having the PufX coding gene, forms only monomers and can grow phototrophically. This result indicated that RC-LH1 dimer formation is not only dependent on the presence or absence of PufX protein but also on some unidentified factors (Crouch and Jones, 2012). Mutational studies on PufX protein C-terminal sequences of *Rba. sphaeroides* reveal the importance of R49, G52 or R53 amino acids and alteration of these sequences prevents RC-LH1dimer formation (Qian et al., 2017).

**FIGURE 4:**
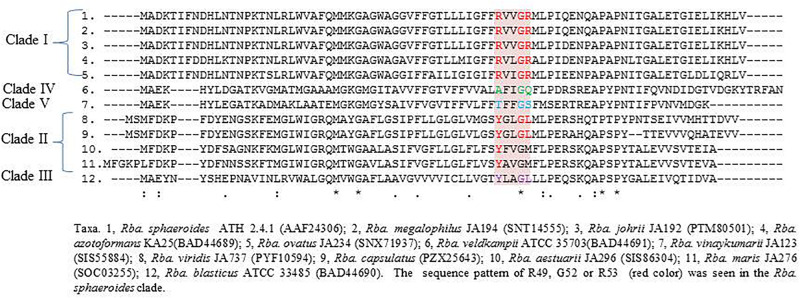
PufX protein sequence alignment using clustalW, showing the R49, G52 or R53 sequence pattern, which is important for RC-LH1 dimer formation.

To analyze members of *Rhodobacter* spp. for the presence of the sequence pattern required for RC-LH1 dimer formation, we extracted the PufX protein sequences of *Rhodobacter* members from NCBI and aligned them using CLUSTALW. Interestingly, all the members of *Rba. sphaeroides* clade have R49, G52 or R53 amino acids (Figure 4), while none of the other members of *Rhodobacter* outside of the *Rba. sphaeroides* clade has such a pattern. A phylogenetic tree (Supplementary Figure S4) constructed using the PufX protein sequences of *Rhodobacter* members and some phylogenetically related chemotrophic bacteria shows that *Rhodobacter* spp. are polyphyletic.

#### Photosynthetic Gene Cluster (PGC)

**FIGURE 5:**
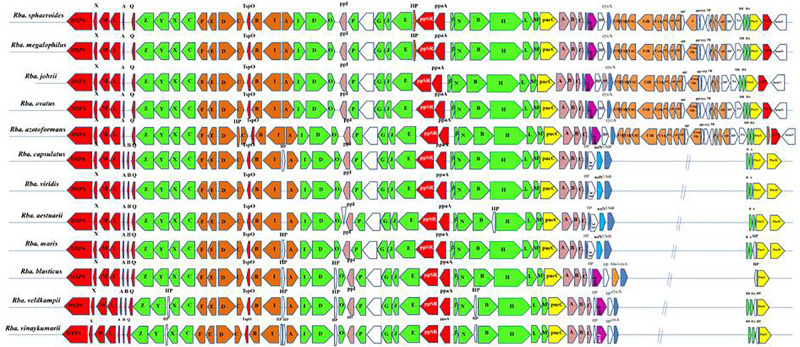
Arrangement of photosynthetic gene cluster and structure in *Rhodobacter*. Green, bacterial chlorophyll genes; red, *puf* and regulators genes; pink, *puh* genes; orange, carotenoid genes; blue, *hem* and *cyc* gene; yellow, *lha*A gene; blank, uncertain or unrelated genes; gray, hypothetical protein.

All essential genes required for photosynthesis are organized in a continuous stretch called the photosynthetic gene cluster (PGC). Phylogenetic analysis of one or two photosynthetic genes (Imhoff et al., 2018) is not sufficient to study the photosynthetic gene phylogeny among the members of the ‘Rhodobacteraceae’ (Brinkmann et al., 2018). Thus, in addition to the PufLM or PufX protein based phylogenetic analysis, we studied the photosynthetic gene arrangement in the PGC to differentiate *Rhodobacter* spp. Comparative analysis of the PGC of *Rhodobacter* spp. (Figure 5) revealed that members of the *Rba. sphaeroides* clade lack the *pufB* gene sequence encoding for light-harvesting antenna LH1-beta subunit. In contrast the *acsF* gene encoding for the Mg-protoporphyrin IX oxidative cyclase, aerobic form, was not found in the genomes of members of the *Rba. capsulatus* clade (Figure 5). In the *Rba. sphaeroides* clade, urea metabolism genes were seen after the *cycA gene*, whereas in the remaining clades these genes are scattered elsewhere in their genomes but not adjacent to the PGC. The PGC gene encoded protein sequences were concatenated and used to construct a phylogenetic tree (Supplementary Figure S5), which was congruent with the PufLM protein, PufX protein and 16S rRNA gene phylogenetic trees. In the PGC based phylogenetic tree *Rhodobacter* spp. formed four monophyletic clades (Supplementary Figure S5).

### Polar Lipid Analyses

The polar lipid composition of *Rhodobacter* spp. typically includes phosphatidylethanolamine, phosphatidylglycerol, uncharacterized aminolipid, phosphatidylcholine (except *Rba. veldkampii*) and other unidentified lipids. In addition, clade I members have DPG and an unidentified glycolipid (GL). Polar lipid profiles of the representative strains are shown in Supplementary Figure S6.

## Discussion

The use of genomics in taxonomic studies improves the credibility, reliability and reproducibility of the data, serves as an informative tool in establishing phylogenetic relationships among prokaryotes and helps in eliminating unreliable methods and subjective difficult-to-replicate data (Chun and Rainey, 2014; Chun et al., 2018; Pérez-Cataluña et al., 2018). To resolve the taxonomic conflict which have arisen due to heterogeneity among the *Rhodobacter* spp., genome based analysis was carried out in this study. The genome size of clade I members is relatively larger than that of the remaining clades, with the exception of *Rba. ovatus*. The clade I members have higher G+C content (68.2–69.1 mol%) when compared with clade II (61.0–66.6 mol%), clade III (66.4–66.5 mol%), clade IV (65.0 mol%) and clade V (68.2 mol%). According to Nouioui et al. (2018) genome size variation can be a genus specific taxonomic marker, indicating that the clade I members might be belong to a distinct genus from *Rhodobacter sensu stricto* (Clade II). This is also in agreement with the separation of *Rba. capsulatus* strains from *Rba. sphaeroides* in the phylogenomic analysis of Simon et al. (2017).

Taxonomy of bacteria based on core- and pan-genome analysis is a powerful extension of the polyphasic approach (Inglin et al., 2018). Pan-genome analysis of the genus *Rhodobacter* revealed that only 1239 core genes are present (Supplementary Table S4). An increased number of core genes was observed when the clade wise pan genome analysis performed, which indicated the inter cluster genomes were divergent.

Digital DDH values (Supplementary Table S5) among *Rhodobacter* members support the current species delineations, except between *Rba. sphaeroides* 2.4.1^T^ and *Rba. megalophilus* JA194^T^ which exceeds the cut off of 70% *d*DDH value for prokaryotic species delineation (Wayne et al., 1984). This suggests that *Rba. megalophilus* JA194^T^ can be a sub-species of *Rba. sphaeroides* ATH 2.4.1^T^. With the advancement in next generation sequencing methods, ANI is increasingly being used to delineate closely related species (Kim et al., 2014). Bacterial strains having less than 95–96% of ANI are considered as distinct species (Goris et al., 2007; Richter and Rosselló-Móra, 2009; Rodriguez and Konstantinidis, 2014). In agreement with the *d*DDH data, OrthoANI values also confirm that all species of *Rhodobacter* are well described at species level, exception for *Rba. megalophilus* which gave an ANI score of 97.96% with *Rba. sphaeroides* 2.4.1^T^ (Supplementary Table S5), confirming these two strains belong to the same species.

Qin et al. (2014) proposed that if the POCP values between two species were less than 50% then they belong to two different genera. However, this threshold does not appear to apply among members of *Rhodobacter* (Supplementary Table S6), suggesting that they all belong to a single genus. POCP cut off values are ineffective in delineating genera in the family ‘Rhodobacteraceae’ (Wirth and Whitman, 2018), *Methylococcaceae* (Orata et al., 2018) and *Neisseriaceae* (Li et al., 2017) suggesting a need to establish appropriate POCP cut-off values for different families.

**FIGURE 6:**
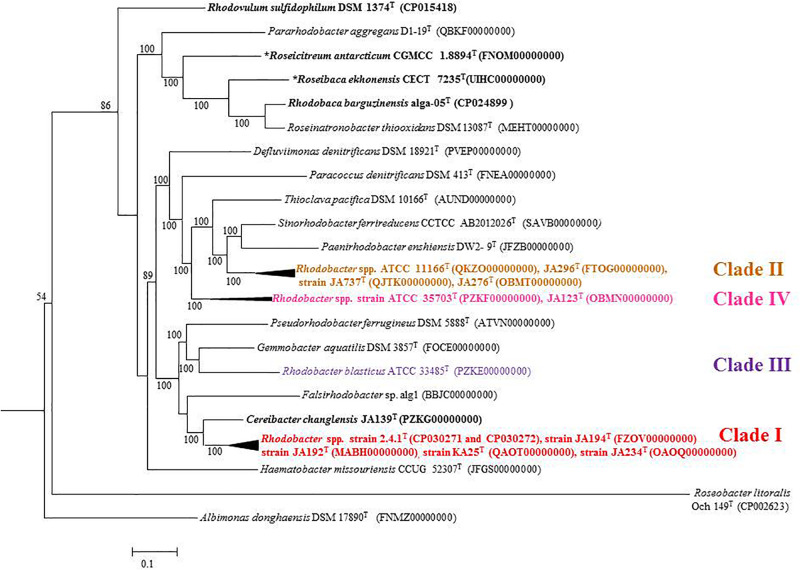
Phylogenomic tree of type strains of genus *Rhodobacter* and its nearest member with *Escherichia coli* and *Pseudomonas* as the out group. The tree was elucidated using the FastME from the whole proteome. The numbers above branches are pseudo-bootstrap support values from 100 replicates, only values above 50% are shown. Phototrophic bacteria are indicated by bold letters and aerobic anoxygenic phototrophic bacteria with bold letters and star.

Percentage of AAI below 60% between the compared genomes of species was also proposed to delineate genera (Rodriguez and Konstantinidis, 2014). However, the AAI values between members of related but different genera can vary between 60 and 80% (Luo et al., 2014; Orata et al., 2018). The similarity index of AAI between *Rhodobacter* spp. (Supplementary Table S6) strengthen the proposal for reclassifying them, while the phylogenetically related genera (chemotrophs) in the family ‘Rhodobacteraceae’ are well described and require no further reclassification. The phylogenomic tree topology using genomic DNA sequences (UBCG; Figure 3) or protein sequences (Figure 6 and Supplementary Figure S7) was in congruence with the 16S rRNA gene-based phylogeny (Figure 1). The phylogenetic tree based on the proteins (Supplementary Figures S3–S5) involved in photosynthesis also showed the polyphyletic assemblage of the *Rhodobacter* spp. which indicates the need of reclassification of some members of the genus *Rhodobacter.*

The conflict observed in the current classification of the genus *Rhodobacter* is visible in single gene based trees, either in the 16S rRNA gene (Figure 1) or *rpoB* gene based tree (Gandham et al., 2018), which is strengthened by better resolved whole genome phylogenies. The results of phylogenomic analysis emphasize the need for reclassification of the taxonomically complex genus *Rhodobacter.* Housekeeping genes (such as *rpoB*) can be used to determine the phylogenetic relationships of species when the 16S rRNA gene sequence based phylogenetic tree is unable to resolve the phylogenetic position of taxa, based on the fact that the house keeping genes are less conversed and evolve faster than the 16S rRNA gene (Martens et al., 2008). Based on the 16S rRNA gene and *rpoB* gene phylogenetic trees we believe that genome sequences for the remaining four members (*Rba. alkalitolerans*, *Rba. azollae, Rba. lacus* and *Rba. sediminis*) are not essential to resolve the taxonomic positions of members of the genus *Rhodobacter*.

### Phenotypic and Chemotaxonomic Characters Between the Clades

The members of *Rba. sphaeroides* clade (clade I) were isolated from freshwater environments only (Srinivas et al., 2008; Girija et al., 2010; Gandham et al., 2018), whereas *Rba. capsulatus* clade (clade II) members have been isolated from freshwater (Suresh et al., 2017), estuarine (Venkata Ramana et al., 2009) and marine habitats (Venkata Ramana et al., 2008). *Rba. blasticus*, which is assigned to clade III, and *Rba. veldkampii* (clade IV member), were isolated from freshwater habitats (Eckersley and Dow, 1980; Hansen and Imhoff, 1985), while *Rba. vinaykumarii* was isolated from a marine habitat (Srinivas et al., 2007). However, the isolation source is not the distinguishing feature between the clades. The members of clade II are motile (Suresh et al., 2017), whereas some of the members of clade I do not exhibit motility, and members of clade III, IV, and clade V are not motile (Eckersley and Dow, 1980; Hansen and Imhoff, 1985; Srinivas et al., 2007). Except for *Rba. blasticus* which multiplies by sessile budding and has a lamellar ICM, the remaining species of *Rhodobacter* multiply by binary fission and have a vesicular architecture of their ICMs (Table 1). Only the clade V member (*Rba. vinaykumarii* JA123^T^) has a requirement of NaCl for optimum growth and cannot assimilate sulfate (Srinivas et al., 2007). Only one member (*Rhodobacter azotoformans*) of clade I has denitrification ability (Hiraishi et al., 1996).

**TABLE 1:** Differentiating characters of taxa of the genus *Rhodobacter* and closely related genera of the family ‘Rhodobacteraceae.’

**Characteristic**	**1**	**2**	**3**	**4**	**5**	**6**	**7**	**8**	**9**	**10**	**11**	**12**	**13**	**14**
Cell shape	R-O	R-O	R-O	R-O	R	R-O	R	R-O	R	R,O	R	R-O	R	C-R
Motility	±	+	–	–	–	–	±	±	–	±	–	±	±	±
Phototrophic growth	+	+	+	+	+	+	–	–	–	–	–	+	–	–
Multiplication	Binary fission	Binary fission	Sessile budding	Binary fission	Binary fission	Binary fission	Binary fission	Binary fission/Budding	nd	nd	Binary fission	Binary fission	nd	Binary fission
Slime production	±	±	–	–	+	+	–	nd	nd	nd	nd	±		nd
Growth at 5°C	±	–	–	–	–	+	–	±	–	–	+	nd	–	±
Vitamins	b,n,t	b,n,t	n,t,b	b,paba,t	b	b,n,t	–	b,n,t,paba,B_3_,B_12_	nd	nd	b,n,t,paba,B_3_	b,n,t,paba, B_12_, complex	B_3_,B_12_	nd
NaCl requirement	–	–	–	–	+	–	+	±	+	+	–	±	–	+
Sulfate assimilation	+	+	+	–	+	+	nd	nd	nd	nd	nd	±	–	–
Denitrification	±	–	–	–	–	–	–	±	–	±^∗^	–	–	–	±
BChl-*a*	+	+	+	+	+	+	–	–	–	–	–	+	–	–
IMA	Vesicles	Vesicles	Lamellar	Vesicles	Vesicles	Vesicles	–	–	–	–	–	Vesicles	–	–
Production of carotenoids	+	+	+	+	+	+	–	–	–	–	–	+	–	–
Light harvesting complexes	LH1,LH2	LH1,LH2	LH1,LH2	LH1,LH2	LH1,LH2	LH1,LH2	–	–	–	–	–	LH1,LH2	–	–
Photoautotrophic growth	±	±	+	+	–	–	–	–	–	–	–	±	–	–
Dark anaerobic growth	±	±	–	nd	–	+	–	–	–	–	–	±	–	–
**Fatty acids**														
C_18__:0_3OH	–	+	nd	–	–	–	–	+	–	±	–	–	–	±
C_18__:__1_ω7c11 methyl	+	–		–	+	+	±	±	–	±	–	+	+	±
**Polar lipids**														
GL	+	–	–	–	–	+	+	± –	–	–	–	–	nd	+
SL	–	–	–	–	–	–	–	–	–	–	–	+		–
PC	+	+	+	–	+	+	+	+	–	+	–	–		+
DPG	+	–	–	–	–	+	+	±	–	–	–	–		+
Isolation source	Fresh, soil	Fresh and Marine	Freshwater	Freshwater	Marine	Snow sample	Soil, Slattern	Freshwater	Blood, Nose	Marine	Soil	Hyper-saline and marine	Marine	Soil, sewage

*(1) *Rhodobacter sphaeroides* clade (*Rba*. *sphaeroides, Rba*. *johrii*, *Rba*. *megalophilius, Rba*. *azotoformans*, *Rba*. *ovatus* and *Rba*. *alkalitolerans*; data from this study, Hiraishi et al., 1996; Imhoff, 2005; Arunasri et al., 2008; Srinivas et al., 2008; Girija et al., 2010; Gandham et al., 2018); (2) *Rhodobacter sensu stricto* clade (*Rba. capsulatus, Rba. aestuarii, Rba. maris*, *Rba. viridis*, *Rba. sediminis, Rba. lacus* and *Rba. azollae*; data from this study and from Imhoff, 2005; Venkata Ramana et al., 2009; Girija et al., 2010; Shalem Raj et al., 2013; Subhash and Lee, 2016; Suresh et al., 2017); (3) *Rhodobacter blasticus* clade (*Rba. blasticus*; data from this study, data taken from Eckersley and Dow, 1980; Girija et al., 2010); (4) *Rhodobacter veldkampii* clade (*Rba. veldkampii;* data from this study, data taken from Hansen and Imhoff, 1985; Girija et al., 2010); (5) *Rba*. *vinaykumarii* clade (*Rba*. *vinaykumarii*; data from this study, data taken from Srinivas et al., 2007; Girija et al., 2010); (6) *Cereibacter* (*Cer. changlensis*; data taken from Girija et al., 2010; Suresh et al., 2015); (7) *Falsirhodobacter* (*F. deserti*, *F. halotolerans*; data taken from Subhash et al., 2013; Wang et al., 2015); (8) *Gemmobacter* (10 species; data taken from Rothe et al., 1987; Chen C.X. et al., 2013; Sheu et al., 2013; Liu et al., 2014; Kämpfer et al., 2015; Suresh et al., 2015); (9) *Haematobacter* (*H. missouriensis*, *H. massiliensis*; data taken from Helsel et al., 2007; Subhash et al., 2013; Wang et al., 2014); (10) *Pseudorhodobacter* (four strains, data taken from Uchino et al., 2002; Jung et al., 2012; Chen W.M. et al., 2013; Lee et al., 2013; Zhang et al., 2016); (11) *Paenirhodobacter* (*P. enshiensis*; data taken from Wang et al., 2014); (12) *Rhodovulum* (based on nineteen species including the recently described *Rdv. aestuarii*; data taken from Lakshmi et al., 2011; Srinivas et al., 2012, 2014; Nupur et al., 2014; Divyasree et al., 2015; Suresh et al., 2015); (13) *Pararhodobacter* (*P. aggregans*; data taken from Foesel et al., 2011; Xie et al., 2015); (14) *Paracoccus* (based on 41 species; data taken from Kämpfer et al., 2012; Sun et al., 2013; Pan et al., 2014; Zhang et al., 2014). R-O, rod to oval; R, rod; C-R, coccoid to rod; b, Biotin; B_12_, vitamin B_12_; B_3_, vitamin B_3_; n, niacin; paba, p-aminobenzoic acid; t, thiamine; PC, Phosphatidylcholine; SL, Sulfolipid; GL, Glycolipid; DPG, Diphosphatidylglycerol; LH, light harvesting system; +, present; −, absent; ^∗^, data of nitrate reduction; nd, No data available; IMA, Internal membrane architecture.*

All the members of clade I (*Rba*. *sphaeroides*, *Rba*. *johrii*, *Rba. megalophilus*, *Rba. azotoformans*, *Rba. ovatus* and *Rba. alkalitolerans*) have glycolipid and DPG (Gandham et al., 2018; Supplementary Figure S6). Polar lipid analysis has been a central tool in chemotaxonomy and the presence/absence of glycolipid was considered as one of the genus differentiating characters in the reclassification of some species of the genus *Rhodobium* into a new genus, *Afifella* (Urdiain et al., 2008). Members of clade II, III, IV, and V do not have DPG or glycolipid (Suresh et al., 2017; Supplementary Figure S6). Except for some of members of clade II, all members of the new genera proposed below have spheroidene as their major carotenoid (Girija et al., 2010; Suresh et al., 2017; Gandham et al., 2018). The chemotaxonomic comparison between the genus *Rhodobacter sensu stricto*, the proposed novel genera and closely related genera is given in the Table 1.

### Justification for Separating *Rhodobacter* spp. Into New Genera

Phenotypic and phylogenomic features support the separation of genus *Rhodobacter* into four different genera. Emended descriptions and reclassifications are given for these taxa. Apart from the tree topologies, with interspersing chemotrophic members, the branch lengths in the whole-genome and 16S rRNA trees indicated that the *Rhodobacter* species are too divergent to be placed into the same genus. Since its description, five out of 21 originally proposed *Rhodobacter* species have been reclassified into different genera and it has been emphasized that *Rhodobacter* is still heterogeneous and should be subdivided based on additional molecular taxonomic data (Hiraishi and Ueda, 1994; Lee et al., 2005; Helsel et al., 2007; Suresh et al., 2015).

16S rRNA gene and *rpoB* gene (Gandham et al., 2018) based phylogenetic trees shows that *Rba. alkalitolerans* JA916^T^ together with *Rba. johrii*, *Rba. megalophilus, Rba. sphaeroides*, *Rba. azotoformans* and *R. ovatus* fall outside of the *Rba. capsulatus* clade and belong in what we define as the *Rba*. *sphaeroides* clade. We propose that the members of the *Rba*. *sphaeroides* clade be classified into a new genus for which we propose the name *Luteovulum* gen. nov. Moreover, as *Rba. sphaeroides* 2.4.1^T^ and *Rba*. *megalophilus* JA194^T^ have high genomic indices (OrthoANI and *d*DDH) we conclude that they are not two different species. Based on phenotypic differences (Arunasri et al., 2008), we propose *Rba. megalophilus* as a sub-species of *Rba. sphaeroides*.

*Rba. viridis, Rba. azollae*, *Rba. sediminis, Rba. capsulatus, Rba. aestuarii, Rba. lacus* and *Rba. maris* form a distinct clade in both the single gene trees (*rpoB* gene and 16S rRNA gene). This clade accommodates the type species of the genus *Rhodobacter*, *Rba. capsulatus* and members of this clade are proposed to be included in the genus *Rhodobacter sensu stricto*. *Rba. veldkampii* and *Rba. vinaykumarii* formed two different clusters with interspersing chemotrophs. However, in the genome-based trees (UBCG based, proteome based) they are clustered together. Genome based phylogenomic trees have better resolution than single gene-based trees, so for the time being we are not separating these two into two different genera, which can be done in future based on multiple species/strains. For the *Rba. veldkampii* clade accommodating *Rba. vinaykumarii* and *Rba. veldkampii*, we propose the new genus *Phaeovulum* gen. nov. To accommodate *Rba. blasticus*, we propose the new genus *Fuscovulum* gen. nov. The classification of *Rba. blasticus* itself controversial when it was transferred from the genus *Rhodopseudomonas* to *Rba. blasticus*, despite having lamellar ICM structures in contrast to all other members of genus *Rhodobacter*, which have vesicular ICM structures. It was proposed that ICM systems may be of questionable value as genus specific characters, because they are not coincident with the 16S rRNA gene phylogenetic relationships (Kawasaki et al., 1993). Later Hiraishi and Ueda (1994) suggested separating *Rba. veldkampii* and *Rba. blasticus* from the genus *Rhodobacter* into a novel genus based on the ICM architecture, mode of cell division of *Rba. blasticus*, and final oxidation product of sulfide in the case of *Rba. veldkampii*. Hiraishi and Ueda (1994) considered that mode of cell division and ICM types are genus specific characters. Here we propose the reclassification of each of these species into separate genera which, after two and half decades, helps to resolve the taxonomic conflict within the genus *Rhodobacter*, based on the genomic and chemotaxonomic information.

### Emended Description of the Genus *Rhodobacter* (Imhoff et al., 1984) Srinivas et al. (2007)

Members can be isolated from freshwater ponds, estuarine and marine environments. Oval-to-rod shaped cells have vesicular ICM architecture. Cells are mostly motile with a single polar flagellum and multiply by binary fission. Catalase and oxidase positive. Primarily phototrophic and contain BChl-*a* and carotenoids of the spheroidene series. Aerobic growth occurs in most species. Mesophilic. Phototrophic growth occurs on a range of organic substrates. The growth factors biotin, niacin and thiamine, alone or in combination, are required for growth to occur. NaCl requirement is not obligatory, can tolerate up to 2–3%. C_18__:__1_ω7c/C_18__:__1_ω6C, C_18__:__0_, C_16__:__0_ and C_16__:__1_ω7C/C_16__:__1_ω6c are the major fatty acids. Most species have C_10__:0_3OH and C_18__:0_3OH as fatty acid alcohols. Phosphatidylethanolamine, phosphatidylglycerol and phosphatidylcholine are the major polar lipids. Glycolipid and diphosphatidylglycerol are absent. Hopanoids are not produced. Q-10 is the major quinone.

The type species is *Rhodobacter capsulatus* (Molisch, 1907) Imhoff et al. (1984).

### Taxonomic Note on *Rhodobacter megalophilus* Arunasri et al. (2008)

Based on the OrthoANI and *d*DDH analysis, *Rba. megalophilus* is not a distinct species as the differences between *Rba. sphaeroides* and *Rba. megalophilus* represent intra-species divergence. It should be noted that *Rba. sphaeroides* and *Rba. megalophilus* differ with regard to growth at 5°C, absence of flagellar motility, cell suspension color, vitamins required for growth, ability to utilize sulfide or hydrogen as electron donors and ability to utilize citrate as carbon source (Arunasri et al., 2008). Based on chemotaxonomic data we propose that *Rba. megalophilus* be reclassified as a sub-species of *Rba. sphaeroides*.

### Description of *Luteovulum* gen. nov.

*Luteovulum* [Lu.te.o’vu.lum. L. adj. *luteus* yellow; N.L. dim. neut. n. *ovulum* (from L. n. *ovum*, an egg) a small egg; N.L. neut. n. *Luteovulum* small yellow egg].

Members can be isolated from freshwater ponds, paddy soils, wastewater treatment plants, alkaline ponds and lake sediments. Gram-stain negative, oval-to-rod shaped cells and have vesicular ICM architecture. Cells are mostly motile with a single polar flagellum and multiply by binary fission. Catalase and oxidase positive. Primarily phototrophic and contain BChl-*a* and carotenoids of the spheroidene series. Facultative aerobes and mesophilic. Phototrophic growth occurs on a number of organic substrates. The growth factors biotin, niacin and thiamine, alone or in combination, are required for growth. NaCl requirement is not obligatory, can tolerate up to 2–3%. C_18__:__1_ω7c/C_18__:__1_ω6C, C_18__:__0_, C_16__:__0_, C_10__:__0_ 3OH, C_16__:__1_ω7C/C_16__:__1_ω6c, C_18__;__1_ω7c11 methyl are the major fatty acids. Phosphatidylethanolamine, phosphatidylglycerol, diphosphatidylglycerol, phosphatidylcholine and an unidentified glycolipid are the major polar lipids. Hopanoids are not produced. Q10 is the major quinone. Delineation of the genus is based on 16S rRNA and *rpoB* gene-based phylogeny, phylogenomics, genome comparison and chemotaxonomic differences.

The type species is *Luteovulum sphaeroides* subsp. *sphaeroides.*

### Description of *Luteovulum sphaeroides* subsp. *sphaeroides* subsp. nov.

The description of *Luteovulum sphaeroides* is identical to that of *Rba. sphaeroides* (Imhoff et al., 1984; Imhoff, 2005) except for the following modifications. C_17__:__0_ is present in minor quantities. An unidentified aminolipid, an unidentified phospholipid and two unidentified lipids are additional polar lipids. Type strain is available from the DSMZ (DSM 158^T^) and LMG (LMG 2827^T^). The 16S rRNA gene sequence GenBank/EMBL/DDBJ accession number of the type strain is X53853 and CP030271, CP030272, CP030273, CP030274, CP030275, and CP030276 are the genome sequence accession numbers of the type strain.

### Description of *Luteovulum sphaeroides* subsp. *megalophilum* subsp. nov.

*Luteovulum megalophilum* (me.ga.lo’phi.lum. Gr. adj. *megas*, wide; N.L. adj. *philus* -*a* -*um* (from Gr. adj. *philos* -*ê* -*on*) friend, loving; N.L. neut. adj. *megalophilum*, wide (temperature)-loving). The description of *Luteovulum megalophilum* is identical to that of *Rba. megalophilus* (Arunasri et al., 2008) except for the following modifications. 3-Hydroxy C_10__:__0_ and C_12__:__0_ fatty acids are present. The DNA G+C content of the type strain calculated from genome sequence is 68.8 mol%. The type strain is available from JCM (JCM 14598^T^) and KCTC (KCTC 5602^T^). The 16S rRNA gene sequence GenBank/EMBL/DDBJ accession number of the type strain AM421024 and that of the genome sequence is FZOV00000000.

### Description of *Luteovulum johrii* comb. nov.

*Luteovulum johrii* (joh’ri.i. N.L. masc. gen. n. *johrii* of B. N. Johri, an eminent and well-known Indian microbiologist).

**Basonym:** *Rhodobacter johrii* Girija et al. (2010).

The description of *Luteovulum johrii* is identical to that of *Rba. johrii* (Girija et al., 2010). The type strain is available from the JCM (JCM 14543^T^) and DSMZ (DSM 18678^T^). The 16S rRNA gene sequence GenBank/EMBL/DDBJ accession number of the type strain is and that of the genome sequence is MABH00000000.

### Description of *Luteovulum ovatum* comb. nov.

*Luteovulum ovatum* (o.va’tum. L. neut. adj. *ovatum*, egg-shaped, ovate)

**Basonym:** *Rhodobacter ovatus* Srinivas et al. (2008).

The description of *Luteovulum ovatum* is identical to that of *Rba. ovatus* (Srinivas et al., 2008). Type strain is available at JCM (JCM 14779^T^) and CCUG (CCUG 55049^T^). AM690348 is the 16S rRNA gene sequence GenBank/EMBL/DDBJ accession number and OAOQ00000000 is the genome sequence accession number.

### Description of *Luteovulum azotoformans* comb. nov.

*Luteovulum azotoformans* (a.zt.to.for’mans. N.L. n. *azotum* [from French n. azote (from Gr. prep. *a*, not; Gr. n. *zôê*, life; N. Gr. n. *azôê*, not sustaining life)], nitrogen; N.L. pref. *azo*-, pertaining to nitrogen; L. part. adj. *formans*, forming; N.L. part. adj. *azotoformans*, nitrogen forming).

**Basonym:** *Rhodobacter azotoformans* Hiraishi et al. (1996).

The description of *Luteovulum azotoformans* is identical to that of *Rba. azotofarmans* (Hiraishi et al., 1996). The type strain is available from JCM (JCM 9340^T^) and NBRC (NBRC 16436^T^). The 16S rRNA gene sequence GenBank/EMBL/DDBJ accession number of the type strain is D70846 and that of the genome sequence is QAOT00000000.

### Description of *Luteovulum alkalitolerans* comb. nov.

*Luteovulum alkalitolerans* (al.ka.li.to’le.rans. N.L. n. *alkali*, alkali; L. part. adj. *tolerans*, tolerating; N.L. part. adj. *alkalitolerans*, alkali-tolerating).

**Basonym:** *Rhodobacter alkalitolerans* Gandham et al. (2018).

The description of *Luteovulum alkalitolerans* is identical to that of *Rba. alkalitolerans* (Gandham et al., 2018). The type strain is available from the KCTC (KCTC 15473^T^) and LMG (LMG 28749^T^). The 16S rRNA gene sequence GenBank/EMBL/DDBJ accession number of the type strain is LN810645.

### Description of *Fuscovulum* gen. nov.

*Fuscovulum* [Fusc.o’vu.lum. L. masc. adj. *fuscus*, tawny N.L. dim. neut. n. *ovulum* (from L. n. *ovum*, an egg), a small egg; N.L. neut. n. *Fuscovulum* small tawny egg].

Members can be isolated from freshwater habitats. Gram-stain negative, rod-to-oval shaped cells have lamellar ICM architecture. Cells are mostly non-motile and multiply by sessile budding. Catalase and oxidase positive. Primarily phototrophic and contain BChl-*a* and carotenoids of the spheroidene series. Growth occurs under aerobic, anaerobic and mesophilic conditions. Phototrophic growth occurs on a number of organic substrates. Nicotinic acid and thiamine are required for growth. NaCl is not required for growth. Q-10 is the major quinone. The G+C mol% of the type strain of the type species is 66.5%. Delineation of the genus is based on 16S rRNA and *rpoB* gene-based phylogeny, phylogenomics, genome comparison and chemotaxonomic differences.

The type species is *Fuscovulum blasticum.*

### Description of *Fuscovulum blasticum* comb. nov.

*Fuscovulum blasticum* (Gr. adj. *blastikos* -*ê* -*on*, budding, sprouting; N.L. masc. adj. *blasticum*, budding, apt to bud).

**Basonym:** *Rhodobacter blasticus* (Eckersley and Dow, 1980) Kawasaki et al. (1993).

The description of *Fuscovulum blasticum* is identical to that of *Rhodopseudomonas blastica* (Eckersley and Dow, 1980) and *Rhodobacter blasticus* (Imhoff et al., 1984; Imhoff, 2005). The type species is available from the ATCC (ATCC 33485^T^) and NBRC (NBRC 16437^T^). The 16S rRNA gene sequence GenBank/EMBL/DDBJ accession number of the type strain is DQ342322 and that of the genome sequence is PZKE00000000.

### Description of *Phaeovulum* gen. nov.

*Phaeovulum* [Phae.o’vu.lum. N.L. neut. adj. *phaeum* (from Gr. neut. adj. phaion), brown; N.L. dim. neut. n. *ovulum* (from L. n. *ovum*, an egg) a small egg; N.L. neut. n. *Phaeovulum* small brown egg].

Members can be isolated from marine habitats including tidal waters, fresh water. Gram-stain negative, rod to oval shaped cells have vesicular ICM architecture. Cells are mostly non-motile and multiply by binary fission. Catalase and oxidase positive. Primarily phototrophic and contain BChl-*a* and carotenoids of the spheroidene series. Growth occurs under anaerobic and mesophilic conditions. Phototrophic growth occurs on a number of organic substrates. Biotin, *para*-aminobenzoate and thiamine are required for growth either singly or in combination. Some strains do not contain phosphatidylcholine. Some require NaCl for growth and can tolerate up to 4%. C_18__:__1_ω7c/C_18__:__1_ω6C, C_18__:__0_, C_16__:__0_, C_10__:__0_ 3OH, C_16__:__1_ω7C/C_16__:__1_ω6c, C_18__;__1_ω7c11methyl are the major fatty acids. Q-10 is the major quinone. G+C mol% is 65–68.25. Delineation of the genus is based on 16S rRNA and *rpoB* gene based phylogeny, phylogenomics, genome comparison and chemotaxonomic differences.

The type species is *Phaeovulum veldkampii.*

### Description of *Phaeovulum veldkampii* comb. nov.

*Phaeovulum veldkampii* (veld. kamp’i.i. N. L. gen. masc. n. *veldkampii* of Veldkamp; named for Hans Veldkamp, a Dutch microbiologist).

**Basonym:** *Rhodobacter veldkampii* Hansen and Imhoff (1985).

The description of *Phaeovulum veldkampii* is identical to that of *Rhodobacter veldkampii* (Hansen and Imhoff, 1985; Imhoff, 2005). The type strain is available from the ATCC (ATCC 35703^T^) and DSMZ (DSM 11550^T^). The 16S rRNA gene sequence GenBank/EMBL/DDBJ accession number is D16421 and that of the genome sequence is PZKF00000000.

### Description of *Phaeovulum vinaykumarii* comb. nov.

*Phaeovulum vinaykumarii* (vi’nay.ku.ma’ri.i. N.L. masc. gen. n. *vinaykumarii* of Vinaykumar, named after the late Dr. M. Vinaykumar, an Indian microbiologist and research supervisor of CVR and CS, who initiated work on anoxygenic phototrophic bacteria in India).

**Basonym:** *Rhodobacter vinaykumarii* Srinivas et al. (2007).

The description of *Phaeovulum vinaykumarii* is identical to that of *Rhodobacter vinaykumarii* (Srinivas et al., 2007). The type strain is available from the JCM (JCM 14544^T^) and DSMZ (DSM 18714^T^). The 16S rRNA gene sequence GenBank/EMBL/DDBJ accession number of the type strain is AM408117 and that of the genome sequence is OBMN00000000.

## Data Availability Statement

The raw data supporting the conclusions of this manuscript will be made available by the authors, without undue reservation, to any qualified researcher.

## Author Contributions

CVR and CS designed the work. GS maintained the bacterial cultures. TL and GS genomic data retrieval from databases, pangenomic, phylogenomic, and phylogenetic data analysis. GS, TL, and BI calculated the genomic relatedness indices. All authors made the manuscript and accepted the work for publication.

## Conflict of Interest

The authors declare that the research was conducted in the absence of any commercial or financial relationships that could be construed as a potential conflict of interest.

## Footnotes

1 http://www.bacterio.net/

2 http://www.bacterio.net/rhodobacteraceae.html

3 https://patricbrc.org

4 https://www.ebi.ac.uk/Tools/psa/lalign/

## References

[B1] Aliyu H. Lebre P. Blom J. Cowan D. De Maayer P. (2016). Phylogenomic re-assessment of the thermophilic genus *Geobacillus*. *Syst. Appl. Microbiol.* 39 527–533. 10.1016/j.syapm.2016.09.004 27726901

[B2] Arunasri K. Ramana V. V. Spröer C. Sasikala Ch. Ramana Ch. V. (2008). *Rhodobacter megalophilus* sp. nov., a phototroph from the Indian Himalayas possessing a wide temperature range for growth. *Int. J. Syst. Evol. Microbiol.* 58 1792–1796. 10.1099/ijs.0.65642-0 18676458

[B3] Auch A. F. Klenk H. P. Göker M. (2012). Standard operating procedure for calculating genome-to-genome distances based on high-scoring segment pairs. *Stand. Genomic. Sci.* 2 142–148. 10.4056/sigs.541628 21304686 PMC3035261

[B4] Breider S. Scheuner C. Schumann P. Fiebig A. Petersen J. Pradella S. (2014). Genome-scale data suggest reclassifications in the *Leisingera-Phaeobacter* cluster including proposals for *Sedimentitalea* gen. nov. and *Pseudophaeobacter* gen. nov. *Front. Microbiol.* 5:416. 10.3389/fmicb.2014.00416 25157246 PMC4127530

[B5] Brinkmann H. Göker M. Koblížek M. Wagner-Döbler I. Petersen J. (2018). Horizontal operon transfer, plasmids, and the evolution of photosynthesis in *Rhodobacteraceae*. *ISME J.* 12 1994–2010. 10.1038/s41396-018-0150-9 29795276 PMC6052148

[B6] Chaudhari N. M. Gupta V. K. Dutta C. (2015). BPGA- an ultra-fast pan-genome analysis pipeline. *Sci. Rep.* 6:24373. 10.1038/srep24373 27071527 PMC4829868

[B7] Chen W. M. Cho N. T. Huang W. C. Young C. C. Sheu S. Y. (2013). Description of *Gemmobacter fontiphilus* sp. nov., isolated from a freshwater spring, reclassification of *Catellibacterium nectariphilum* as *Gemmobacter nectariphilus* comb. nov., *Catellibacterium changlense* as *Gemmobacter changlensis* comb. nov., *Catellibacterium aquatile* as *Gemmobacter aquaticus nom.* nov., *Catellibacterium caeni* as *Gemmobacter caeni* comb. nov., *Catellibacterium nanjingense* as *Gemmobacter nanjingensis* comb. nov., and emended description of the genus *Gemmobacter* and of *Gemmobacter aquatilis*. *Int. J. Syst. Evol. Microbiol.* 63 470–478. 10.1099/ijs.0.042051-0 22493172

[B8] Chen C. X. Zhang X. Y. Liu C. Yu Y. Liu A. Li G. W. (2013). *Pseudorhodobacter antarcticus* sp. nov., isolated from Antarctic intertidal sandy sediment, and emended description of the genus *Pseudorhodobacter* Uchino et al., 2002 emend. Jung et al., 2012. *Int. J. Syst. Evolut. Microbiol.* 63 849–854. 10.1099/ijs.0.042184-0 22611201

[B9] Chun J. Oren A. Ventosa A. Christensen H. Arahal D. R. da Costa M. S. (2018). Proposed minimal standards for the use of genome data for the taxonomy of prokaryotes. *Int. J. Syst. Evol. Microbiol.* 68 461–466. 10.1099/ijsem.0.002516 29292687

[B10] Chun J. Rainey F. A. (2014). Integrating genomics into the taxonomy and systematics of the Bacteria and Archaea. *Int. J. Syst. Evol. Microbiol.* 64 316–324. 10.1099/ijs.0.054171-0 24505069

[B11] Crouch L. Jones M. (2012). Cross-species investigation of the functions of the *Rhodobacter* PufX polypeptide and the composition of the RC–LH1 core complex. *Biochim. Biophys. Acta* 2 336–352. 10.1016/j.bbabio.2011.10.009 22079525

[B12] Divyasree B. Lakshmi K. V. N. S. Bharti D. Sasikala Ch. Ramana Ch. V. (2015). *Rhodovulum aestuarii* sp. nov., isolated from a brackish waterbody. *Int. J. Syst. Evol. Microbiol.* 66 165–171. 10.1099/ijsem.0.000691 26475698

[B13] Eckersley K. Dow C. S. (1980). *Rhodopseudomonas blastica* sp. nov.: a member of the Rhodospirillaceae. *J. Gen. Microbiol.* 119 465–473. 10.1099/00221287-119-2-465

[B14] Foesel B. Drake H. Schramm A. (2011). *Defluviimonas denitrificans* gen. nov., sp. nov., and *Pararhodobacter aggregans* gen. nov., sp. nov., non-phototrophic *Rhodobacteraceae* from the biofilter of a marine aquaculture. *Syst. Appl. Microbiol.* 34 498–502. 10.1016/j.syapm.2011.08.006 21959289

[B15] Gandham S. Lodha T. Sasikala C. Ramana C. V. (2018). *Rhodobacter alkalitolerans* sp. nov., isolated from an alkaline brown pond. *Arch. Microbiol.* 200 1487–1492. 10.1007/s00203-018-1561-8 30167725

[B16] Girija K. R. Sasikala Ch. Ramana Ch. V. Sproer C. Takaichi S. Thiel V. (2010). *Rhodobacter johrii* sp. nov., an endospore producing cryptic species isolated from semi-arid tropical soils. *Int. J. Syst. Evol. Microbiol.* 60 2099–2107. 10.1099/ijs.0.011718-0 19854875

[B17] Goris J. Konstantinidis K. T. Klappenbach J. A. Coenye T. Vandamme P. Tiedje J. M. (2007). DNA-DNA hybridization values and their relationship to whole-genome sequence similarities. *Int. J. Syst. Evol. Microbiol.* 57 81–91. 10.1099/ijs.0.64483-0 17220447

[B18] Gupta R. S. Lo B. Son J. (2018). Phylogenomics and comparative genomic studies robustly support division of the genus *Mycobacterium* into an emended genus *Mycobacterium* and four novel genera. *Front. Microbiol.* 9:67. 10.3389/fmicb.2018.00067 29497402 PMC5819568

[B19] Hansen T. A. Imhoff J. F. (1985). *Rhodobacter veldkampii*, a new species of phototrophic purple nonsulfur bacteria. *Int. J. Syst. Bacteriol.* 35 115–116. 10.1099/00207713-35-1-115 26263629

[B20] Helsel L. O. Hollis D. Steigerwalt A. G. Morey R. E. Jordan J. Aye T. (2007). Identification of “*Haematobacter*,”a new genus of aerobic Gram-negative rods isolated from clinical specimens, and reclassification of *Rhodobacter massiliensis* as “*Haematobacter massiliensis* comb. nov.”. *J. Clin. Microbiol.* 45 1238–1243. 10.1128/jcm.01188-06 17287332 PMC1865840

[B21] Hiraishi A. Muramatsu K. Ueda Y. (1996). Molecular genetic analyses of *Rhodobacter azotoformans* sp. nov. and related species of phototrophic bacteria. *Syst. Appl. Microbiol.* 19 168–177. 10.1016/s0723-2020(96)80042-7

[B22] Hiraishi A. Ueda Y. (1994). Intrageneric structure of the genus *Rhodobacter*: transfer of *Rhodobacter sulfidophilus* and related marine species to the genus *Rhodovulum* gen. nov. *Int. J. Syst. Bacteriol.* 44 15–23. 10.1099/00207713-44-1-15

[B23] Imhoff J. F. (2005). “Genus *Rhodobacter*,” in *Bergey’s Manual of Systematic Bacteriology*, 2nd Edn, vol. 2, eds Brenner D. J. Krieg N. R. Staley J. T. Garrity G. M. (New York, NY: Springer), 161–167.

[B24] Imhoff J. F. Rahn T. Künzel S. Neulinger S. C. (2018). Photosynthesis is widely distributed among *Proteobacteria* as demonstrated by the phylogeny of PufLM reaction center proteins. *Front. Microbiol.* 8:2679. 10.3389/fmicb.2017.02679 29472894 PMC5810265

[B25] Imhoff J. F. Trüper H. G. Pfennig N. (1984). Rearrangement of the species and genera of the phototrophic ‘purple non sulfur bacteria’. *Int. J. Syst. Bacteriol.* 34 340–343. 10.1099/00207713-34-3-340

[B26] Inglin R. C. Meile L. Stevens M. J. A. (2018). Clustering of pan- and core-genome of *Lactobacillus* provides novel evolutionary insights for differentiation. *BMC Genomics* 19:284. 10.1186/s12864-018-4601-5 29690879 PMC5937832

[B27] Jung Y. T. Ooh K. H. Oh T. K. Yoon J. H. (2012). *Pseudorhodobacter aquimaris* sp. nov. isolated from sea water, and emended description of the genus *Pseudorhodobacter* Uchino et al., 2002. *Int. J. Syst. Evol. Microbiol.* 62 100–105. 10.1099/ijs.0.029769-0 21335494

[B28] Kates M. (1972). “Techniques in lipidology,” in *Laboratory Techniques in Biochemistry and Molecular Biology*, Vol. 3 eds Work T. S. Work E. (New York, NY: American Elsevier Publishing Company), 355–356.

[B29] Kates M. (1986). “Techniques of lipidology: isolation, analysis and identification of lipids,” in *Laboratory Techniques in Biochemistry and Molecular Biology*, Vol. 3 eds Burdon R. H. van Knippenberg P. H. (Amsterdam: Elsevier), 100–112.

[B30] Kawasaki H. Hoshino Y. Hirata A. Yamasato K. (1993). Is intracytoplasmic membrane structure a generic criterion? It does not coincide with phylogenetic interrelationships among phototrophic purple non-sulfur bacteria. *Arch. Microbiol.* 160 358–362. 8257281 10.1007/BF00252221

[B31] Kim M. Oh H. S. Park S. C. Chun J. (2014). Towards a taxonomic coherence between average nucleotide identity and 16S rRNA gene sequence similarity for species demarcation of prokaryotes. *Int. J. Syst. Evol. Microbiol.* 64 346–351. 10.1099/ijs.0.059774-0 24505072

[B32] Kimura M. (1980). A simple method for estimating evolutionary rate of base substitutions through comparative studies of nucleotide sequences. *J. Mol. Evol.* 16 111–120. 10.1007/bf01731581 7463489

[B33] Kumar S. Stecher G. Tamura K. (2016). MEGA7: molecular evolutionary genetics analysis version 7.0 for bigger datasets. *Mol. Biol. Evol.* 33 1870–1874. 10.1093/molbev/msw054 27004904 PMC8210823

[B34] Kämpfer P. Jerzak L. Wilharm G. Golke J. Büsse H. Glaeser S. (2015). *Gemmobacter intermedius* sp. nov., isolated from a white stork (*Ciconia ciconia*). *Int. J. Syst. Evol. Microbiol.* 65 778–783. 10.1099/ijs.0.000012 25479954

[B35] Kämpfer P. Lai W. A. Arun A. B. Young C. C. Rekha P. D. Martin K. (2012). *Paracoccus rhizosphaerae* sp. nov., isolated from the rhizosphere of the plant Crossostephium chinense (L.) Makino (Seremban). *Int. J. Syst. Evol. Microbiol.* 62 2750–2756. 10.1099/ijs.0.039057-0 22286908

[B36] Lakshmi K. V. N. S. Sasikala Ch. Takaichi S. Ramana Ch. V. (2011). *Phaeospirillum oryzae* sp. nov., a spheroplast forming phototrophic alphaproteobacterium from a paddy soil. *Int. J. Syst. Evol. Microbiol.* 61 1656–1661. 10.1099/ijs.0.025544-0 20709914

[B37] Lee K. B. Liu C. T. Anzai Y. Kim H. Aono T. Oyaizu H. (2005). The hierarchical system of the “*Alphaproteobacteria*”: description of *Hyphomonadaceae fam.* nov., *Xanthobacteraceae fam.* nov. and *Erythrobacteraceae fam.* nov. *Int. J. Syst. Evol. Microbiol.* 55 1907–1919. 10.1099/ijs.0.63663-0 16166687

[B38] Lee M. Lee S. Jung Y. Park S. Yoon J. J. (2013). *Pseudorhodobacter wandonensis* sp. nov., isolated from wood falls, and emended description of the genus Pseudorhodobacter. *Int. J. Syst. Evol. Microbiol.* 63 1479–1484. 10.1099/ijs.0.042879-0 22843726

[B39] Li Y. Xue H. Sang S. Q. Lin C. L. Wang X. Z. (2017). Phylogenetic analysis of family *Neisseriaceae* based on genome sequences and description of *Populibacter corticis* gen. nov., sp. nov., a member of the family Neisseriaceae, isolated from symptomatic bark of Populus euramericana canker. *PLoS One* 12:e0174506. 10.1371/journal.pone.0174506 28406911 PMC5390963

[B40] Liu J. J. Zhang X. Q. Chi F. T. Pan J. Sun C. Wu M. (2014). *Gemmobacter megaterium* sp. nov., isolated from coastal planktonic seaweeds. *Int. J. Syst. Evol. Microbiol.* 64 66–71. 10.1099/ijs.0.050955-0 24014623

[B41] Lopes-Santos L. Castro D. B. A. Ferreira-Tonin M. Corrêa D. B. A. Weir B. S. Park D. (2017). Reassessment of the taxonomic position of *Burkholderia andropogonis* and description of *Robbsia andropogonis* gen. nov., comb. nov. *Antonie van Leeuwenhoek* 110 727–736. 10.1007/s10482-017-0842-6 28190154

[B42] Luo C. Rodriguez-R L. M. Konstantinidis K. T. (2014). MyTaxa: an advanced taxonomic classifier for genomic and metagenomic sequences. *Nucleic Acids Res.* 42:e73. 10.1093/nar/gku169 24589583 PMC4005636

[B43] Martens M. Dawyndt P. Coopman R. Monique G. Vos P. D. Willems A. (2008). Advantages of multilocus sequence analysis for taxonomic studies: a case study using 10 housekeeping genes in the genus Ensifer (including former *Sinorhizobium*). *Int. J. Syst. Evol. Microbiol.* 58 200–214. 10.1099/ijs.0.65392-0 18175710

[B44] Meier-Kolthoff J. P. Göker M. (2019). TYGS is an automated high-throughput platform for state-of-the-art genome-based taxonomy. *Nat. Commun.* 10:2182. 10.1038/s41467-019-10210-3 31097708 PMC6522516

[B45] Molisch H. (1907). *Die Purpurbakterien Nach Neuen Untersuchungen.* Jena: G. Fischer.

[B46] Na S. I. Kim Y. O. Yoon S. H. Ha S. M. Baek I. Chun J. (2018). UBCG: up-to-date bacterial core gene set and pipeline for phylogenomic tree reconstruction. *J. Microbiol.* 56 281–285. 10.1007/s12275-018-8014-6 29492869

[B47] Nouioui I. Carro L. Garcia-Lopez M. Meier-Kolthoff J. P. Woyke T. Kyrpides N. C. (2018). Genome-based taxonomic classification of the phylum *Actinobacteria*. *Front. Microbiol.* 9:2007. 10.3389/fmicb.2018.02007 30186281 PMC6113628

[B48] Nupur P. Srinivas T. N. R. Takaichi S. Anil K. P. (2014). *Rhodovulum mangrove* sp. nov. a phototrophic alphaproteobacterium isolated from a mangrove forest sediment sample. *Int. J. Syst. Evol. Microbiol.* 64 3168–3173. 10.1099/ijs.0.059857-0 24972612

[B49] Orata F. D. Meier-Kolthoff J. P. Sauvageau D. Stein L. Y. (2018). Phylogenomic analysis of the gamma*proteobacteria*l methanotrophs (Order Methylococcales) calls for the reclassification of members at the genus and species levels. *Front. Microbiol.* 9:3162. 10.3389/fmicb.2018.03162 30631317 PMC6315193

[B50] Oren A. Duker S. Ritter S. (1996). The polar lipid composition of Walsby’s square bacterium. *FEMS. Microbiol. Lett.* 138 135–140. 10.1016/0378-1097(96)00085-7

[B51] Pan J. Sun C. Zhang X. Huo Y. Zhu X. Wu M. (2014). *Paracoccus sediminis* sp. nov., isolated from Pacific Ocean marine sediment. *Int. J. Syst. Evol. Microbiol.* 64 2512–2516. 10.1099/ijs.0.051318-0 24812365

[B52] Pérez-Cataluña A. Collado L. Salgado O. Lefiñanco V. Figueras M. (2018). A polyphasic and taxogenomic evaluation uncovers *Arcobacter cryaerophilus* as a species complex that embraces four genomovars. *Front. Microbiol.* 9:805. 10.3389/fmicb.2018.00805 29755434 PMC5934430

[B53] Pujalte M. J. Lucena T. Ruvira M. A. Arahal D. R. Macián M. C. (2014). “The family *Rhodobacteraceae*,” in *The Prokaryotes*, eds Rosenberg E. DeLong E. F. Lory S. Stackebrandt E. Thompson F. (Berlin: Springer), 439–512. 10.1007/978-3-642-30197-1_377

[B54] Qian P. Martin E. C. Ng I. W. Hunter C. N. (2017). The C-terminus of PufX plays a key role in dimerisation and assembly of the reaction center light-harvesting 1 complex from *Rhodobacter sphaeroides*. *Biochim. Biophys. Acta Bioenerg.* 1858 795–803. 10.1016/j.bbabio.2017.06.001 28587931 PMC5538271

[B55] Qin Q. Xie B. Zhang X. Chen X. Zhou B. Zhou J. (2014). A proposed genus boundary for the prokaryotes based on genomic insights. *J. Bacteriol.* 196 2210–2215. 10.1128/jb.01688-14 24706738 PMC4054180

[B56] Richter M. Rosselló-Móra R. (2009). Shifting the genomic gold standard for the prokaryotic species definition. *Proc. Natl. Acad. Sci. U.S.A.* 106 19126–19131. 10.1073/pnas.0906412106 19855009 PMC2776425

[B57] Rodriguez L. M. Konstantinidis K. T. (2014). Bypassing cultivation to identify bacterial species. *Microbe.* 9 111–117.

[B58] Rosselló-Móra R. Amann R. (2015). Past and future species definitions for Bacteria and Archaea. *Syst. Appl. Microbiol.* 38 209–216. 10.1016/j.syapm.2015.02.001 25747618

[B59] Rothe B. Fischer A. Hirsch P. Sittig M. Stackebrandt E. (1987). The phylogenetic position of the budding bacteria Blastobacter aggregates and *Gemmobacter aquatilis* gen. nov., sp. nov. *Arch. Microbiol.* 147 92–99. 10.1007/bf00492911

[B60] Shalem Raj P. Ramaprasad E. V. V. Vaseef S. Sasikala Ch. Ramana Ch. V. (2013). *Rhodobacter viridis* sp. nov., a phototrophic bacterium isolated from mud of a stream. *Int. J. Syst. Evol. Microbiol.* 63 181–186. 10.1099/ijs.0.038471-0 22389279

[B61] Sheu S. Y. Sheu D. S. Sheu F. S. Chen W. M. (2013). *Gemmobacter tilapiae* sp. nov., a poly-β-hydroxybutyrate accumulating bacterium isolated from a freshwater culture pond. *Int. J. Syst. Evol. Microbiol.* 63 1550–1556. 10.1099/ijs.0.044735-0 22888190

[B62] Simon M. Scheuner C. Meier-Kolthoff J. P. Brinkhoff T. Wagner-Döbler I. Ulbrich M. (2017). Phylogenomics of *Rhodobacteraceae* reveals evolutionary adaptation to marine and non-marine habitats. *ISME J.* 11 1483–1499. 10.1038/ismej.2016.198 28106881 PMC5437341

[B63] Srinivas A. Rahul K. Ramprasad E. V. V. Sasikala Ch. Ramana Ch. V. (2012). *Rhodovulum bhavnagarense* sp. nov., a phototrophic alphaproteobacterium isolated from a pink pond. *Int. J. Syst. Evol. Microbiol.* 62 2528–2532. 10.1099/ijs.0.036152-0 22180610

[B64] Srinivas A. Vinay Kumar B. Divya Sree B. Tushar L. Sasikala Ch. Ramana Ch. V. (2014). *Rhodovulum salis* sp. nov. and *Rhodovulum viride* sp. nov., phototrophic Alphaproteobacteria isolated from marine habitats. *Int. J. Syst. Evol. Microbiol.* 64 957–962. 10.1099/ijs.0.058974-0 24425825

[B65] Srinivas T. N. R. Anil Kumar P. Sasikala Ch. Ramana Ch. V. Imhoff J. F. (2007). *Rhodobacter vinaykumarii* sp. nov., a marine phototrophic alphaproteobacterium from tidal waters, and emended description of the genus Rhodobacter. *Int. J. Syst. Evol. Microbiol.* 57 1984–1987. 10.1099/ijs.0.65077-0 17766859

[B66] Srinivas T. N. R. Anil Kumar P. Sasikala Ch. Spröer C. Ramana Ch. V. (2008). *Rhodobacter ovatus* sp. nov., an alphaproteobacterium isolated from industrially polluted freshwater pond. *Int. J. Syst. Evol. Microbiol.* 58 1379–1383. 10.1099/ijs.0.65619-0 18523181

[B67] Subhash Y. Lee S. S. (2016). *Rhodobacter sediminis* sp. nov. isolated from lagoon sediments. *Int. J. Syst. Evol. Microbiol.* 66 2965–2970. 10.1099/ijsem.0.001130 27150292

[B68] Subhash Y. Tushar L. Sasikala Ch. Ramana Ch. V. (2013). *Falsirhodobacter halotolerans* gen. nov., sp. nov., isolated from dry soils of a solar saltern. *Int. J. Syst. Evol. Microbiol.* 63 2132–2137. 10.1099/ijs.0.044107-0 23104358

[B69] Sun L. N. Zhang J. Kwon S. W. He J. Zhou S. G. Li S. P. (2013). *Paracoccus huijuniae* sp. nov., an amide pesticide-degrading bacterium isolated from activated sludge of a wastewater biotreatment system. *Int. J. Syst. Evol. Microbiol.* 63 1132–1137. 10.1099/ijs.0.044180-0 22753523

[B70] Suresh G. Sailaja B. Ashif A. Dave B. P. Sasikala Ch Ramana Ch. V. (2017). Description of *Rhodobacter azollae* sp. nov. and *Rhodobacter lacus* sp. nov. *Int. J. Syst. Evol. Microbiol.* 67 3289–3295. 10.1099/ijsem.0.002107 28829020

[B71] Suresh G. Sasikala Ch. Ramana Ch. V. (2015). Reclassification of *Gemmobacter changlensis* to a new genus as *Cereibacter changlensis* gen. nov., comb. nov. *Int. J. Syst. Evol. Microbiol.* 65 794–798. 10.1099/ijs.0.000016 25481291

[B72] Tindall B. J. (1990b). Lipid composition of *Halobacterium lacusprofundi*. *FEMS. Microbiol. Lett.* 66 199–202. 10.1016/j.bbamem.2016.08.010 27565574

[B73] Tindall B. J. (1990a). A comparative study of the lipid composition of *Halobacterium saccharovorum* from various sources. *Syst. Appl. Microbiol.* 13 128–130. 10.1016/s0723-2020(11)80158-x

[B74] Uchino Y. Hamada T. Yokota A. (2002). Proposal of *Pseudorhodobacter ferrugineus* gen. nov., comb. nov., for a non-photosynthetic marine bacterium, *Agrobacterium ferrugineum*, related to the genus Rhodobacter. *J. Gen. Appl. Microbiol.* 48 309–320. 12682869 10.2323/jgam.48.309

[B75] Urdiain M. López-López A. Gonzalo C. Busse H. J. Langer S. Kämpfer P. (2008). Reclassification of *Rhodobium marinum* and *Rhodobium pfennigii* as *Afifella marina* gen. nov. comb. nov. and *Afifella pfennigii* comb. nov., a new genus of photoheterotrophic Alphaproteobacteria and emended descriptions of Rhodobium, Rhodobium orientis and Rhodobium gokarnense. *Syst. Appl. Microbiol.* 31 339–351. 10.1016/j.syapm.2008.07.002 18774253

[B76] Venkata Ramana V. Kumar A. P. Srinivas T. N. R. Sasikala Ch. Ramana Ch. V. (2009). Rhodobacter aestuarii sp. nov., a phototrophic alphaproteobacterium isolated from an estuarine environment. *Int. J. Syst. Evol. Microbiol.* 59 1133–1136. 10.1099/ijs.0.004507-0 19406806

[B77] Venkata Ramana V. Sasikala Ch. Ramana Ch. V. (2008). *Rhodobacter maris* sp. nov., a phototrophic alphaproteobacterium isolated from a marine habitat of India. *Int. J. Syst. Evol. Microbiol.* 58 1719–1722. 10.1099/ijs.0.65638-0 18599723

[B78] Wang D. Liu H. Zheng S. Wang G. (2014). *Paenirhodobacter enshiensis* gen. nov., sp. nov., a non-photosynthetic bacterium isolated from soil, and emended descriptions of the genera Rhodobacter and Haematobacter. *Int. J. Syst. Evol. Microbiol.* 64 551–558. 10.1099/ijs.0.050351-0 24135316

[B79] Wang L. Zhou Z. Wu G. Chen M. Lin M. Zhang W. (2015). *Falsirhodobacter deserti* sp. nov., isolated from sandy soil. *Int. J. Syst. Evol. Microbiol.* 65 650–655. 10.1099/ijs.0.068262-0 25424484

[B80] Wayne L. G. Brenner D. J. Colwell R. R. Grimont P. A. D. Kandler O. Krichevsk M. I. (1984). International committee on systematic bacteriology. Report of the ad hoc committee on reconciliation of approaches to bacterial systematics. *Int J. Syst. Bacteriol.* 37 463–464.

[B81] Wirth J. S. Whitman W. B. (2018). Phylogenomic analyses of a clade within the roseobacter group suggest taxonomic reassignments of species of the genera *Aestuariivita*, *Citreicella*, *Loktanella*, *Nautella*, *Pelagibaca*, *Ruegeria*, *Thalassobius*, *Thiobacimonas* and *Tropicibacter*, and the proposal of six novel genera. *Int. J. Syst. Evol. Microbiol.* 68 2393–2411. 10.1099/ijsem.0.002833 29809121

[B82] Xie B. S. Lv X. L. Cai M. Tang Y. Q. Wang Y. N. Cui H. L. (2015). *Plastorhodobacter daqingensis* gen. nov., sp. nov.: a non-phototrophic Bacterium isolated from Daqing Oilfield. *Curr. Microbiol.* 70 657–664. 10.1007/s00284-014-0769-3 25572494

[B83] Yoon S. H. Ha S. M. Lim J. M. Kwon S. J. Chun J. (2017). A large-scale evaluation of algorithms to calculate average nucleotide identity. *Antonie van Leeuwenhoek* 110 1281–1286. 10.1007/s10482-017-0844-4 28204908

[B84] Zhang G. Yang Y. Yin X. Wang S. (2014). *Paracoccus pacificus* sp. nov., isolated from the Western Pacific Ocean. *Antonie van Leeuwenhoek.* 106 725–731. 10.1007/s10482-014-0242-0 25086778

[B85] Zhang Y. Jiang F. Chang X. Qiu X. Ren L. Qu Z. (2016). *Pseudorhodobacter collinsensis* sp. nov., isolated from a till sample of an icecap front. *Int. J. Syst. Evol. Microbiol.* 66 178–183. 10.1099/ijsem.0.000693 26476707

[B86] Zuckerkandl E. Pauling L. (1965). “Evolutionary divergence and convergence in proteins,” in *Evolving Genes and Proteins*, eds Bryson V. Vogel H. J. (New York, NY: Academic Press), 97–166. 10.1016/b978-1-4832-2734-4.50017-6

