# Corrigendum: Taxogenomics Resolves Conflict in the Genus *Rhodobacter*: A Two and Half Decades Pending Thought to Reclassify the Genus *Rhodobacter*

**DOI:** 10.3389/fmicb.2020.01111

**Published:** 2020-06-03

**Authors:** G. Suresh, Tushar D. Lodha, B. Indu, Ch. Sasikala, Ch. V. Ramana

**Affiliations:** ^1^Department of Plant Sciences, School of Life Sciences, University of Hyderabad, Hyderabad, India; ^2^Bacterial Discovery Laboratory, Centre for Environment, Institute of Science and Technology, Jawaharlal Nehru Technological University Hyderabad, Hyderabad, India

**Keywords:** *Rhodobacter*, taxogenomics, *Rhodobacter sensu stricto*, gen. nov., *Rhodobacter* reclassification, phylogenomics, proposal of 3 new phototrophic genera, photosynthetic gene cluster

In the original article, there was an error in the proposal of *Luteovulum sphaeroides* subsp*. sphaeroides* as the type species of *Luteovulum* gen. nov. This is because according to Rule 15 of the International Code of Nomenclature of Prokaryotes (Parker et al., 2019), the type of a genus is a designated species. The correct descriptions of *Luteovulum* gen. nov. and species belonging to the genus *Luteovulum* are given below, with *Luteovulum sphaeroides* comb. nov. proposed as type species. The corrections have been made to the following sub-sections of the **Discussion** section:

**Description of** ***Luteovulum* gen. nov**.

*Luteovulum* (Lu.te.o'vu.lum. L. masc. adj. *luteus* yellow; N.L. dim. neut. n. *ovulum*, a small egg; N.L. neut. n. *Luteovulum* small yellow egg).

Members can be isolated from freshwater ponds, paddy soils, wastewater treatment plants, alkaline ponds and lake sediments. Gram-stain negative, oval-to-rod shaped cells and have vesicular ICM architecture. Cells are mostly motile with a single polar flagellum and multiply by binary fission. Catalase and oxidase positive. Primarily phototrophic and contain BChl-*a* and carotenoids of the spheroidene series. Facultative aerobes and mesophilic. Phototrophic growth occurs on a number of organic substrates. The growth factors biotin, niacin and thiamine, alone or in combination, are required for growth. NaCl requirement is not required for growth; can tolerate up to 2–3% NaCl. C_18:1_ω7c/C_18:1_ω6C, C_18:0_, C_16:0_, C_10:0_ 3OH, C_16:1_ω7C/C_16:1_ ω6c, C_18;1_ω7c11 methyl are the major fatty acids. Phosphatidylethanolamine, phosphatidylglycerol, diphosphatidylglycerol, phosphatidylcholine and an unidentified glycolipid are the major polar lipids. Hopanoids are not produced. Q10 is the major quinone. Delineation of the genus is based on 16S rRNA and *rpoB* gene-based phylogeny, phylogenomics, genome comparison and chemotaxonomic differences.

The type species is Luteovulum *sphaeroides*.

**Description of** ***Luteovulum sphaeroides* comb. nov**.

***Luteovulum sphaeroides***(sphae.ro'i.des. L. fem. n. *sphaera*, sphere, globe; L. suff. -*oides* (from Gr. suff. -*eides*, from Gr. n. *eidos*, that which is seen, form, shape, figure), resembling, similar; N.L. neut. adj. *sphaeroides*, spherical).

**Basonym:** *Rhodobacter sphaeroides* (van Niel 1944) Imhoff et al. 1984

The description of *Luteovulum sphaeroides* is identical to that of *Rba. sphaeroides* (Imhoff et al., 1984; Imhoff, 2005) except for the following modifications. C_17:0_ is present in minor quantities. An unidentified aminolipid, an unidentified phospholipid and a few unidentified lipids are additional polar lipids. Growth can occur at 5°C. Some strains are non-motile.

The type strain is ATH2.4.1^T^ (= DSM 158^T^ = LMG 2827^T^).

**Description of** ***Luteovulum sphaeroides* subsp**. ***sphaeroides* subsp. nov**.

*Luteovulum sphaeroides* subsp. *sphaeroides* (sphae.ro'i.des. L. fem. n. *sphaera*, sphere, globe; L. suff. -*oides* (from Gr. suff. -*eides*, from Gr. n. *eidos*, that which is seen, form, shape, figure), resembling, similar; N.L. neut. adj. *sphaeroides*, spherical).

The description of *Luteovulum sphaeroides* subsp. *sphaeroides* is identical to that of *Rba. sphaeroides* (Imhoff et al., 1984; Imhoff, 2005) except for the following modifications. C_17:0_ is present in minor quantities. An unidentified aminolipid, an unidentified phospholipid and two unidentified lipids are additional polar lipids.

The type strain is ATH 2.4.1^T^ (= DSM158^T^ = LMG 2827^T^). The 16S rRNA gene sequence GenBank/EMBL/DDBJ accession number of the type strain is X53853. The complete genome sequence accession numbers of the type strain are CP030271, CP030272, CP030273, CP030274, CP030275, and CP030276.

**Description of** ***Luteovulum sphaeroides* subsp**. ***megalophilum* subsp. nov**.

*Luteovulum megalophilum* (me.ga.lo.phi'lum. Gr. adj. *megas*, wide; N.L. adj. *philus*-*a*-*um* (from Gr. adj. *philos*-ê -*on*) friend, loving; N.L. neut. adj. *megalophilum*, wide (temperature)-loving).

The description of *Luteovulum sphaeroides* subsp. *megalophilum* is identical to that of *Rba. megalophilus* (Arunasri et al., 2008) except for the following modifications. 3-Hydroxy C_10:0_ and C_12:0_ fatty acids are present. The DNA G+C content of the type strain calculated from the genome sequence is 68.8 mol%.

The type strain is JA194^T^ (= JCM 14598^T^ = KCTC 5602^T^). The 16S rRNA gene sequence GenBank/EMBL/DDBJ accession number of the type strain is AM421024 and that of the genome sequence is FZOV00000000.

**Description of** ***Luteovulum johrii* comb. nov**.

*Luteovulum johrii* (joh'ri.i. N.L. masc. gen. n. *johrii* of B. N. Johri, an eminent and well-known Indian microbiologist).

**Basonym**: *Rhodobacter johrii* Girija et al. 2010

The description of *Luteovulum johrii* is identical to that of *Rba. johrii* (Girija et al., 2010).

The type strain, *Luteovulum johrii* JA192^T^, is available from the JCM (JCM 14543^T^) and DSMZ (DSM 18678^T^). The 16S rRNA gene sequence GenBank/EMBL/DDBJ accession number of the type strain is AM398152 and that of the genome sequence is MABH00000000.

**Description of** ***Luteovulum ovatum* comb. nov**.

*Luteovulum ovatum* (o.va'tum. L. neut. adj. *ovatum*, egg-shaped, ovate)

**Basonym**: *Rhodobacter ovatus* Srinivas et al. 2008

The description of *Luteovulum ovatum* is identical to that of *Rba. ovatus* (Srinivas et al., 2008).

The type strain is JA234^T^ (= JCM 14779^T^ = CCUG 55049^T^). The GenBank/EMBL/DDBJ accession number of the 16S rRNA gene sequence of the type strain is AM690348 and that of the genome sequence is OAOQ00000000.

**Description of** ***Luteovulum azotoformans* comb. nov**.

*Luteovulum azotoformans* (a.zo.to.for'mans. N.L. n. *azotum* [from French n. azote (from Gr. prep. *a*, not; Gr. n. *zôê*, life; N. Gr. n. *azôê*, not sustaining life)], nitrogen; N.L. pref. *azo*-, pertaining to nitrogen; L. part. adj. *formans*, forming; N.L. part. adj. *azotoformans*, nitrogen forming).

**Basonym**: *Rhodobacter azotoformans* Hiraishi et al. 1997

The description of *Luteovulum azotoformans* is identical to that of *Rba. azotofarmans* (Hiraishi et al., 1996).

The type strain is KA25^T^ (= JCM 9340^T^ = NBRC 16436^T^). The GenBank/EMBL/DDBJ accession number of the 16S rRNA gene sequence of the type strain is D70846 and that of the genome sequence is QAOT00000000.

**Description of** ***Luteovulum alkalitolerans* comb. nov**.

*Luteovulum alkalitolerans* (al.ka.li.to'le.rans. N.L. n. *alkali*, alkali; L. part. adj. *tolerans*, tolerating; N.L. part. adj. *alkalitolerans*, alkali-tolerating).

**Basonym**: *Rhodobacter alkalitolerans* Gandham et al. 2019

The description of *Luteovulum alkalitolerans* is identical to that of *Rba. alkalitolerans* (Gandham et al., 2018).

The type strain is JA916^T^ (= KCTC 15473^T^ = LMG 28749^T^). The 16S rRNA gene sequence GenBank/EMBL/DDBJ accession number of the type strain is LN810645.

The authors apologize for this error and state that this does not change the scientific conclusions of the article in any way. The original article has been updated.
