# Erratum: Corrigendum: Taxogenomics Resolves Conflict in the Genus *Rhodobacter*: A Two and Half Decades Pending Thought to Reclassify the Genus *Rhodobacter*

**DOI:** 10.3389/fmicb.2020.01733

**Published:** 2020-07-09

**Authors:** Frontiers Production Office

**Affiliations:** Frontiers Media SA, Lausanne, Switzerland

**Keywords:** *Rhodobacter*, taxogenomics, *Rhodobacter sensu stricto*, gen. nov., *Rhodobacter reclassification*, phylogenomics, proposal of 3 new phototrophic genera, photosynthetic gene cluster

To enable validation of the names of some of the newly proposed taxa described in this article, the original article should not have been updated following the publication of the corrigendum. The original version has now been reinstated.

