# Supplementary material for: Taxogenomics Resolves Conflict in the Genus Rhodobacter: A Two and Half Decades Pending Thought to Reclassify the Genus Rhodobacter
Source: Front Microbiol. 2019 Oct 31;10:2480. doi: 10.3389/fmicb.2019.02480 (PMC6834548; doi:10.3389/fmicb.2019.02480)
Supplement: Supplementary file 1 [file Data_Sheet_1.pdf]

Table. S1. The organisms used in this study, isolation sources and genome accession numbers

Organisms	Source of Isolation	Accession number
Rhodobacter capsulatus DSM 1710^T	Sewage plants, eutrophic ponds	QKZO00000000
Rhodobacter viridis JA737^T	Mud of a stream	QJTK00000000
Rhodobacter maris JA276^T	Marine sediment	OBMT00000000
Rhodobacter aestuarii JA296^T	Estuarine environment	FTOG00000000
Rhodobacter sphaeroides 2.4.1^T	Sewage plants, eutrophic ponds	CP030271 and CP030272
Rhodobacter johrii JA192^T	Rhizosphere soil	MABH00000000
Rhodobacter megalophilus JA194^T	Soil, Himalayas	FZOV00000000
Rhodobacter azotoformans KA25^T	Sludge for waste water treatment	QAOT00000000
Rhodobacter ovatus JA234^T	Polluted pond	OAOQ00000000
Rhodobacter vinaykumarii JA123^T	Seawater	OBMN00000000
Rhodobacter veldkampii ATCC 35703^T	Freshwater	PZKF00000000
Rhodobacter blasticus DSM 2131T	Eutrophic pond	PZKE00000000
Cereibacter changlensis JA139^T	Snow sample	PZKG00000000
Defluviimonas denitrificans DSM 18921^T	Marine aquaculture	PVEP00000000
Falsirhodobacter sp. alg1	Brown algae	BBJC00000000
Haematobacter missouriensis CCUG 52307^T	Septicemic patient	JFGS00000000
Paenirhodobacter enshiensis DW2-9^T	Soil	JFZB00000000
Pseudorhodobacter ferrugineus DSM 5888^T	Seawater of the Baltic Sea	ATVN00000000
Thioclava pacifica DSM 10166^T	Near-shore sulfidic hydrothermal area	AUND00000000
Gemmobacter aquatilis DSM 3857^T	Fresh water	FOCE00000000
Pararhodobacter aggregans DSM 18938^T	Marine aquaculture	QBKF00000000
Rhodobaca barguzinensis alga-05^T	Sediments of a saline	CP024899
Roseicitreum antarcticum CGMCC 1.8894^T	Sandy intertidal sediment	FNOM00000000
Roseinatronobacter thiooxidans DSM 13087^T	Soda lakes	MEHT00000000
Rhodovulum sulfidophilum DSM 1374^T	Sea sediment	CP015418
Albimonas donghaensis DSM 17890^T	Seawater	FNMZ00000000
Paracoccus denitrificans DSM 413^T	Soil	FNEA00000000
Roseobacter litoralis Och 149^T	Seaweed	CP002623
Escherichia coli ATCC 11775^T	Human, urine, cystitis	JMST00000000
Pseudomonas aeruginosa DSM 50071^T	Soil and water	JYLC00000000
Sinorhodobacter ferrireducens CCTCC AB2012026^T	Microbial fuel cell	SAVB00000000
Roseibaca ekhonensis CECT 7235^T	Lake water	UIHC00000000

Table S2. 16S rRNA gene sequence pairwise similarity percentages among the genus *Rhodobacter*

	1	2	3	4	5	6	7	8	9	10	11	12	13	14	15	16
1	100															
2	100	100														
3	99.9	99.9	100													
4	98.1	98.1	98.1	100												
5	98.5	98.5	98.5	98.8	100											
6	98.4	98.4	98.5	97.5	98.1	100										
7	95.6	95.6	95.7	95.4	95.5	95.8	100									
8	95.7	95.7	95.7	94.8	95.0	94.7	95.7	100								
9	95.8	95.8	95.8	94.8	95.1	94.8	95.6	99.5	100							
10	95.4	95.4	95.4	94.4	94.7	94.4	95.3	99.5	99.9	100						
11	94.9	94.9	94.9	93.8	94.1	93.8	94.6	98.9	99.3	99.4	100					
12	95.1	95.1	95.1	94.4	94.8	95.0	94.4	96.8	97.0	96.7	96.2	100				
13	95.0	95.0	94.9	94.0	94.8	94.5	94.9	97.5	97.3	97.1	96.4	97.8	100			
14	93.9	93.9	93.9	92.8	93.8	93.5	93.7	96.5	97.0	97.0	96.9	96.8	98.4	100		
15	94.8	94.8	94.7	94.8	94.7	94.7	94.6	94.8	94.7	94.5	94.0	95.6	95.0	94.5	100	
16	95.9	95.9	95.9	95.0	95.6	95.5	95.2	96.3	96.4	96.1	95.5	97.8	96.5	95.9	95.9	100

1. *Rhodobacter sphaeroides* 2.4.1^{T}; 2- *Rhodobacter megalophilus* JA194^{T}; 3- *Rhodobacter johrii* JA192^{T}; 4- *Rhodobacter azotoformans* KA25^{T}, 5- *Rhodobacter ovatus* JA234^{T}; 6- *Rhodobacter alkalitolerans* JA916^{T}; 7- *Rhodobacter blasticus* DSM 2131^{T}: 8-*Rhodobacter capsulatus* ATCC 11166^{T}; 9- *Rhodobacter viridis* JA737^{T}; 10- *Rhodobacter sediminis* N1^{T}; 11- *Rhodobacter azollae* JA912^{T}; 12- *Rhodobacter aestuarii* JA296^{T}; 13- *Rhodobacter maris* JA276^{T}; 14- *Rhodobacter lacus* JA826^{T}; 15- *Rhodobacter veldkampii* ATCC 35703^{T}; 16- *Rhodobacter vinaykumarii* JA123^{T}.

Table S3. Genomic information of species/strains of genus *Rhodobacter*.

**Organism name**	**Genome size (Mb)**	**Genome GC%**	**No of Genes**	**No. of proteins**
***Rhodobacter sensu stricto* clade**				
Rba. capsulatus DSM 1710^T	3.66	66.5	3495	3346
Strain SB1003	3.87	66.6	3660	3531
Strain DSM 938	3.86	66.3	3742	3593
Strain DE442	3.76	66.6	3593	3446
Strain B6	3.80	66.4	3607	3403
Strain YW1	3.64	66.6	3497	3354
Strain YW2	3.77	66.4	3576	3414
Strain R121	3.76	66.6	3588	3446
Strain Y262	3.84	66.5	3683	3528
Strain B41	3.67	66.5	3480	3331
Strain A52	3.60	66.5	3411	3357
Rba. viridis JA737^T	3.86	65.3	3701	3481
Rba. maris JA276^T	3.83	65	3745	3557
Rba. aestuarii JA296^T	3.84	61	3695	3544
***Rba. sphaeroides* Clade**				
Rba. sphaeroides ATH 2.4.1^T	4.60	68.7	4474	4280
Strain ATCC 17025	4.55	68.2	4547	4280
Strain ATCC 17029	4.48	68.9	4338	4170
Strain KD131	4.71	69.1	4530	4367
Strain MBTLJ-8	4.66	68.7	4580	4374
Strain MBTLJ-13	4.64	68.7	4561	4360
Strain MBTLJ-20	4.65	68.7	4596	4365
Strain EBL0706	4.43	68.3	4387	4024
Strain AB25	4.58	69.1	4489	4279
Strain AB27	4.4	69.0	4381	4084
Strain AB29	4.54	69.0	4502	4192
Strain AB24	4.74	69.0	4692	4389
Strain WS8N	4.41	69.1	4317	4164
Rba. johrii JA192^T	4.51	69.0	4458	4093
Strain CDR-SL7Cii	4.68	69.1	4496	4330
Rba. megalophilus JA194^T	4.86	68.8	4690	4552
Rba. azotoformans KA25^T	4.41	68.4	4369	4112
Strain YLK20	4.32	68.5	4327	3980
Rba. ovatus JA234^T	3.81	66.5	3668	3422
***Rba. veldkampii* Clade**				
Rba. veldkampii DSM 11550^T	3.26	65	3273	3019
Rba. vinaykumarii JA123^T	3.48	68.2	3281	3122
***Rba. blasticus* Clade**				
Rba. blasticus DSM 2131^T	3.58	66.5	3455	3441
Strain 28/5	3.70	66.4	3595	3494

Table S4: BPGA of all 12 species of genus *Rhodobacter*

Organism name		No. of core genes	No. of accessory genes	No. of unique genes	No. of exclusively absent genes
12 species of genus *Rhodobacter*					
	Rba. aestuarii JA296	1239	1655	699	9
	Rba. azotoformans KA25	1239	2233	730	7
	Rba. blasticus ATCC 33485	1239	1127	1113	57
	Rba. capsulatus ATCC11166	1239	1791	421	2
	Rba. johrii JA192	1239	2594	262	34
	Rba. maris JA276	1239	1832	554	5
	Rba. megalophilus JA194	1239	2961	269	0
	Rba. ovatus JA234	1239	1655	588	8
	Rba. sphaeroides ATH 2.4.1	1239	2478	130	3
	Rba. veldkampii ATCC 35703	1239	1127	657	48
	Rba. vinaykumarii JA123	1239	1184	720	26
	Rba. viridis JA737	1239	1776	543	8
Pan-genome analysis of *Rba. sphaeroides* clade					
	Rba. azotoformans KA25	2294	1082	820	66
	Rba. johrii JA192	2294	1508	293	90
	Rba. megalophilus JA194	2294	1827	345	2
	Rba. ovatus JA234	2294	437	748	531
	Rba. sphaeroides ATH 2.4.1	2294	1402	149	30
Pan-genome analysis of *Rba. capsulatus* clade					
	Rba. aestuarii JA296	2225	571	791	130
	Rba. capsulatus ATCC11166	2225	684	537	53
	Rba. maris JA276	2225	665	727	87
	Rba. viridis JA737	2225	668	663	80
Pan-genome analysis of *Rba. veldkampii* clade					
	Rba. vinaykumarii JA123	2014	0	1123	1004
	Rba. veldkampii ATCC 35703	2014	0	1004	1123

Table S5. ANI and *d*DDH values of genus *Rhodobacter* members and some related genera

Rba. capsulatus clade (1–4) *Rba. sphaeroides* clade (5–9)

ANI (upper right triangle); *d*DDH values (lower left triangle)

	1	2	3	4	5	6	7	8	9	10	11	12	13	14	15	16	17	18	19	20	21	22	23	24	25	26	27	28	29	30	31	32
1		88.04	79.5	77.84	73.92	74.13	73.81	74.01	74.13	75.17	75.62	73.7	74.11	74.02	70.97	71.19	76.13	70.75	75.18	74.08	70.31	70.69	72.28	71.26	73.58	71.43	73.78	69.89	64.07	66.21	78.13	71.14
2	33.9		79.38	77.53	73.95	74.09	74.13	74.11	74.03	75.13	75.3	73.56	74.09	73.78	71.05	71.94	75.9	70.78	75.01	73.86	73.49	70.08	71.97	70.9	73.34	71.39	73.38	69.58	64.2	66.29	78.04	70.81
3	22.1	21.9		79.81	73.54	73.9	73.63	73.64	73.49	75.4	74.77	72.68	73.66	73.49	70.59	71.81	75.21	70.67	75.01	73.39	72.81	70.01	71.54	70.48	72.71	71.09	73.1	69.3	64.16	65.59	77.72	70.71
4	20.3	20.2	22		71.84	72.76	72.04	72.11	72.38	68.25	73.62	71.58	71.93	72.21	70.18	70.44	73.73	70.65	73.63	72.56	71.75	69.9	71.15	70.43	71.29	70.2	74.2	69.08	64	65.27	75.43	70.3
5	19.3	19.5	19.1	19.2		94.9	97.96	85.88	81.21	74.95	74.59	74.67	79.25	74.5	72.5	73.13	74	71.63	73.8	75.19	73.91	70.52	72.53	71.28	74.48	72.11	74.05	69.58	63.65	66.04	75.24	71.21
6	19.2	19.2	18.8	20.1	58.9		94.93	86.03	81.28	75.42	74.68	74.4	79.17	74.34	72.74	73.04	74.36	71.44	73.62	75.3	74.16	70.84	72.69	71.08	74.75	72.12	74.24	69.67	63.46	66.27	75.36	71.49
7	19.1	19.3	19.2	19.1	81.6	59		85.75	81.01	75.22	74.89	74.47	78.98	74.52	72.56	73.28	73.99	71.61	74.06	75.65	74.25	70.67	72.72	71.11	74.45	72.3	74.2	69.6	63.41	66.47	74.95	71.24
8	19.6	19.6	19.2	18.7	30.3	30.6	30.4		81.77	75.43	74.62	74.69	78.9	74.23	72.59	73.23	74.16	71.58	73.77	75.28	73.92	70.56	72.78	71.32	74.49	72.45	74.18	69.67	63.97	66.9	75.28	71.85
9	19.1	19.1	18.9	20.1	24.3	24.4	24.2	24.8		75.71	75.41	74.9	78.74	74.44	72.97	73.33	73.63	72.19	73.22	75.99	73.56	71.38	73.31	71.53	73.97	71.85	74.16	69.96	63.76	66.52	74.37	72.11
10	20	19.7	21.2	20.9	20.3	20.6	20.7	20.5	21		76.95	73.9	74.93	74.7	71.49	72.99	74.94	70.65	75.11	74.38	74.28	71.21	73.03	71.52	74.66	72.29	75.1	70.62	64.43	66.66	76.01	72.06
11	19.3	19.3	19.4	18.8	19.2	19.7	20	19.5	19.7	21		74.25	75.09	74.64	71.74	72.56	74.25	71.89	74.02	74.86	73.76	71.48	73.23	72	73.65	71.62	74.47	69.8	64.56	65.97	75.56	71.7
12	19.3	18.9	18.7	18.6	19.4	19.3	19.3	19.3	19.3	19.4	19.1		74.98	73.69	71.74	72.02	72.74	71.88	72.28	75.71	73.55	70.71	72.59	71.57	73.04	71.23	73.64	69.62	64.27	66.45	73.58	71.33
13	19.2	19.1	18.6	18.6	22.1	22.1	22.1	22.1	21.7	20.2	19.4	19.4		74.89	72.91	73.26	74.3	72.54	73.9	76.67	74.48	71.24	73.2	71.85	74.57	72.42	74.34	70.25	64.2	66.71	75.04	71.6
14	18.8	19.3	19.8	18.9	19.3	19.4	19.6	19	19.1	20.1	19.5	18.9	19.2		71.52	72.6	73.67	71.27	73.87	74.18	73.83	70.65	72.75	71.26	73.83	72.08	74.12	70.11	64.11	66.23	74.53	71.57
15	19.7	19.9	19.2	19.3	19.6	19.2	19.3	18.7	19	19.5	18.4	18.9	18.9	19.2		71.07	71.01	70.55	70.82	72.62	71.18	69.65	71.05	70.23	70.69	69.54	71.22	68.81	63.89	65.11	71.57	69.91
16	19.1	19.3	19.4	19.6	19.9	19.5	20.3	19.5	20.7	20.2	19.2	18.8	19.3	13.9	19.3		72.46	69.9	72.22	72.6	72.31	70.08	71.61	70.17	72.13	71.03	73.04	69.16	64.1	66.05	72.59	70.31
17	19.9	20	20	18.7	19.6	19.5	19.5	19.4	19.3	20	19	18.8	19.3	19.1	18.4	19.5		70.07	75.01	73.25	73.38	70.08	71.63	70.56	73.46	71.72	74.43	69.31	64.43	66.74	79.05	70.76
18	18.4	18.4	18.4	17.8	19.6	19.1	18.8	18.5	18.4	18.4	18.1	18.1	17.8	17.7	18.7	18.9	17.7		70.51	72.85	70.57	70	71.38	70.5	70.32	69.07	70.39	69.31	63.29	63.9	70.71	70.42
19	19.4	19.2	19.5	18.9	19.6	19.2	19.6	18.8	18.6	21.4	19.2	18.8	19.1	19.3	18.8	19.3	19.5	18.2		73.36	73.35	70.31	72.11	70.9	73.27	71.77	73.55	70.14	63.93	66.14	76.67	71.13
20	18.9	19	19.1	18.3	19.5	19.5	19.9	19.6	19.8	19.6	19.6	19.5	20.1	19.2	18.3	19.2	19.4	18.4	18.7		73.8	71.13	73.44	71.94	73.02	71.76	73.81	70.04	64.76	66.39	74.25	71.72
21	19.4	19.4	19.6	19.7	19.7	19.6	19.9	19.6	19.6	20.1	20.4	19.2	19.7	20	19.4	19.6	20.2	17.9	20	19.4		71.39	73.37	72.31	73.78	71.57	73.68	69.66	64.39	65.89	74.34	72.03
22	18.8	18.8	19	18.6	19.6	19.6	19.2	19.6	19	19.6	19.3	18.8	18.6	18.4	19.6	21.8	20.8	18.7	18.7	19.2	19.4		72.7	81.52	70.95	69.63	70.73	69.1	64.27	65.65	70.54	74.54
23	19.1	19.6	20	19.5	19.9	19.3	20.2	19	19.9	19.8	20.4	18.9	19.2	19.8	20.4	18.9	19.2	19.7	19.9	19.1	19.2	20.3		73.2	72.46	70.86	72.22	69.96	63.98	65.42	72.14	73.49
24	18.3	18.8	18.3	17.8	18.1	18.1	17.7	18	18.1	18.6	18.5	18.4	18.6	17.9	18	19.1	19	18	18.1	17.9	18.6	24.1	19.4		71.32	69.81	71.12	69.37	63.66	64.98	71.0	75.29
25	19	18.9	19.2	18.9	19.8	19.6	19.5	19.6	19	19.9	18.6	18.7	19.1	18.6	19.6	19.4	19.1	18.1	19.3	18.9	19.2	19.8	19.1	19		72.19	73.59	70.2	64.33	66.66	74.62	71.28
26	19.7	18.9	19.7	18.7	19.3	19.4	19.1	19.5	18.9	19.5	18.9	18.5	19	19	18.7	19.2	19.1	19.3	19.5	18.8	19	20.4	19	18.1	19		72.15	69.02	65.03	66.83	72.11	70.48
27	19.5	19.4	20	19.7	20.1	20.1	20.1	19.7	19.8	20.5	19.9	19.8	19.8	19.6	19.4	20.3	19.7	17.2	19.7	19.5	19.8	20	19.6	18.6	19.7	19.1		69.72	64.35	67.37	75.0	71.36
28	18.2	18.1	19.1	18.6	18.3	17.8	18.1	18.1	18.5	21	18.4	17	18	17.9	19.7	19.5	19.2	18.5	18.8	17.2	19.2	18	19.2	18	18.8	18.7	17.8		63.63	64.77	69.61	69.97
29	16.9	19.4	18.1	18.9	20.6	19.8	20.1	19.9	20.5	20.1	17.9	18.7	20	17.6	20.6	19.7	20.2	19.9	19.2	20.8	19.5	17.9	18.8	19.4	20.8	19.8	19	19.4		65.03	64.72	63.91
30	17.5	17.6	18	19.8	17	16.9	16.9	16.6	17.5	17	17.4	16.7	16.4	16.2	20.7	17.5	17.3	19.2	17	17.5	15.7	21.4	18	17.1	17.8	16.6	19.4	18.4	19.4		66.85	65.87
31	20.8	20.9	20.9	19.1	19.7	19.6	19.7	19.8	18.8	20.2	19.1	19	19.4	19.2	18.8	19.8	22.5	17.9	20	19	19.6	20	19	19	19.3	19.4	19.9	18.3	19.6	16.2		71.06
32	18.3	18.9	18.8	17.9	19.3	18.9	19.4	20	19	19.6	19.4	18	18.2	19.1	18.5	19.3	18	18.1	18.6	18.2	19.4	19.4	21.4	19.4	19.2	18.1	18.8	18.3	19.4	20.8	18.3	

Taxa; 1- *Rhodobacter capsulatus* ATCC 11166^T; 2- *Rhodobacter viridis* JA737^T; 3- *Rhodobacter maris* JA276^T; 4- *Rhodobacter aestuarii* JA296^T; 5- *Rhodobacter sphaeroides* 2.4.1^T; 6- *Rhodobacter johrii* JA192^T; 7- *Rhodobacter megalophilus* JA194^T; 8- *Rhodobacter azotoformans* KA25^T, 9- *Rhodobacter ovatus* JA234^T; 10- *Rhodobacter vinaykumarii* JA123^T; 11- *Rhodobacter veldkampii* ATCC 35703^T; 12- *Rhodobacter blasticus* DSM 213^T, 13- *Cereibacter changlensis* JA139^T; 14- *Defluviimonas denitrificans* DSM 18921^T; 15- *Falsirhodobacter* sp. alg1; 16- *Haematobacter missouriensis* CCUG 52307^T; 17- *Paenirhodobacter enshiensis* DW2-9^T; 18- *Pseudorhodobacter ferrugineus* DSM 5888^T; 19- *Thioclava pacifica* DSM 10166^T; 20- *Gemmobacter aquatilis* DSM 3857^T; 21- *Pararhodobacter aggregans* DSM 18938^T; 22- *Rhodobaca barguzinensis* alga-05^T; 23- *Roseicitreum antarcticum* CGMCC 1.8894^T; 24- *Roseinatronobacter thiooxidans* DSM 13087^T; 25- *Rhodovulum sulfidophilum* DSM 1374^T; 26- *Albimonas donghaensis* DSM 17890^T; 27- *Paracoccus denitrificans* DSM 413^T; 28- *Roseobacter litoralis* Och 149^T; 29- *Escherichia coli* ATCC 11775^T; 30- *Pseudomonas aeruginosa* DSM 50071^T; 31- *Sinorhodobacter ferrireducens* CCTCC AB2012026^T; 32- *Roseibaca ekhonensis* CECT 7235^T. *Rba. sphaeroides* clade member values in red color; *Rba. capsulatus* clade member values in light brown color; *Rba. veldkampii* clade (*Rba. veldkampii* and *Rba. vinaykumarii*) member values in pink color.

Table S6. AAI and POCP values of genus *Rhodobacter* members and some related genera

AAI values

	Rba. capsulatus clade				*Rba. sphaeroides* clade							POCP values																				
	1	2	3	4	5	6	7	8	9	10	11	12	13	14	15	16	17	18	19	20	21	22	23	24	25	26	27	28	29	30	31	32
1		79.94	75.39	72.62	59.57	57.11	57.23	57.94	59.42	66.58	63.18	62.78	55.21	54.54	52.9	54.07	59.9	55.05	61.88	57.97	48.31	50.86	48.18	50.41	55.36	46.27	49.46	46.1	17.81	21.38	62.21	47.7
2	89.21		73.29	72.1	57.19	54.95	54.88	56.16	59.59	64.41	61.68	61.95	54.25	55.32	50.69	52.28	58.54	55.22	61.09	59.3	47.56	50.32	47.58	50.19	52.41	45.71	48.73	46.46	17.36	21.13	61.86	47.64
3	78.54	78.44		75.63	57.57	55.4	57.13	56.85	58.73	68.07	62.81	61.78	54.95	56.94	51.49	52.85	59.08	55.57	63.08	58.91	49.79	50.68	49.96	50.22	54.63	45.93	49.83	46.88	17.74	21.46	61.32	49.1
4	78.07	77.86	82.33		56.24	54.4	54.19	54.83	59.62	66.37	62.59	59.71	52.88	54.97	50.88	52.16	57.27	54.54	63.68	55.7	47.86	50.8	49.52	49.83	54	45.18	47.11	46.18	17.03	19.94	61.52	48.82
5	64.36	63.95	63.88	63.51		85.1	88.23	76.34	69.76	57.99	57.43	61.71	67.73	54.66	53.18	55.01	54.5	55.01	56.2	58.44	50.34	52.29	50.49	51.25	54.96	48.67	51.34	48.35	18.2	23.01	56.07	48.9
6	64.4	63.86	64.09	63.85	96.32		86.11	74.93	68.26	56.66	56.13	59.29	66.73	53.15	50.98	53.37	52.63	53.34	54.38	56.41	49.66	51.84	50.19	50.27	53.53	47.2	50.68	47.29	17.78	22.87	54.67	48.83
7	64.11	63.58	63.92	63.04	98.56	96.39		76.4	66.46	56.81	55.51	59.38	66.89	54.32	50.1	53.82	52.05	53.32	54.83	57.52	50.02	50.39	50.39	49.87	54.24	46.48	51.65	47.66	18.04	23.13	54.5	48.25
8	64.26	64.02	63.99	63.42	86.42	86.47	86.09		70.52	57.02	56.81	59.77	66.48	54.15	50.67	53.95	51.88	54.25	55.15	55.97	48.79	52.15	49.38	52.04	53.9	46.82	49.71	47.97	17.78	22.36	53.88	49.63
9	65.09	64.98	65.18	64.67	80.94	81.05	80.37	81.08		64.35	64.26	62.8	62.08	55.95	52.88	56.39	55.69	57.33	58.42	59.02	49.27	54.28	53.17	53.68	55.24	48.23	49.85	49.03	17.26	21.42	56.09	54.53
10	67.67	66.97	68.33	67.85	65.41	65.44	65.55	65.16	67.33		71.07	61.26	55.03	57.33	53.89	56.23	60.12	57.87	63.83	59.28	51.31	53.25	54.06	52.77	56.93	49.24	50.37	48.44	17.21	21.07	59.71	54.2
11	69.58	69.27	69.4	69.21	67.35	67.59	67.02	67.17	68.46	72.85		61.55	54.97	56.98	53.69	57.27	58.3	59.2	62.63	59.21	50.13	55.58	54.49	54.92	54.93	47.9	49.89	48.42	16.44	20.17	58.08	56.66
12	64.07	63.67	63.91	63.26	67.25	67	66.92	66.75	68	64.04	66.24		62.48	61.23	54.98	55.15	59.04	62.71	58.88	64.84	52.66	53.4	50.99	52.49	54.19	49.03	50.77	50.93	17.7	22.65	58.68	52.11
13	63.61	63.77	63.67	62.88	74.64	74.03	73.68	73.8	74.86	64.87	67.27	67.9		54.92	52.02	54.09	52.76	56.75	54.48	59.12	51.25	51.98	51.59	51.02	51.99	47.25	52.12	49.02	18.22	23.82	54.39	48.99
14	64.19	64.21	64.39	63.38	64.93	64.54	64.72	64.24	65.72	65.47	67.39	64.95	64.96		50.08	52.23	53.38	59.4	59.83	60.92	53.55	51.78	50.33	50.67	50.69	47.74	50.67	53.11	16.82	21.96	57.5	51.08
15	62.53	62.3	62.46	62.46	66.51	66.13	66.18	66.41	67.31	63.37	64.72	64.34	66.91	62.91		60.06	63.92	52.95	53	53.04	44.13	48.74	47.99	48.81	48.29	52.66	47.34	45.72	19.17	21.59	53.15	46.84
16	63.23	62.83	63.14	62.96	65.53	65.07	65.13	65.4	66.54	64.5	66.13	63.42	64.9	63.64	64.62		63.51	52.96	56.6	55.31	50.32	50.17	49.78	50.07	50.89	53.57	55.23	46.02	19.22	25.34	55.85	47.72
17	69.68	69.33	69.38	68.97	63.74	63.6	63.45	63.78	64.22	66.14	67.64	63.38	63.7	63.93	63.2	64.46		53.22	59.05	58.1	48.89	48.4	48.92	48.3	51.5	51.83	51.96	45.75	19.19	23.24	64.21	47.81
18	62.39	62.39	62.48	62.18	66.5	66.19	66.08	66.13	67.71	63.29	65.2	66.34	68.31	64.1	64.56	62.68	61.72		58.1	62.26	52.12	54.09	52.37	54.2	49.3	48.24	47.96	53.71	15.32	20.35	54.17	52.05
19	69.01	68.5	69.2	69.24	63.29	63.03	62.95	63.12	64.07	67.85	69.13	62.45	62.85	64.74	62.08	63.33	67.59	62.25		60.16	52.58	52.34	50.98	53.22	52.94	48.85	51.41	49.02	16.74	21.43	65.2	51.01
20	64.21	64.38	64.28	63.67	68.12	67.96	68.13	67.87	69.67	64.74	67.43	69.1	70.03	65.52	65.7	64.34	64.19	67.75	63.99		55.06	52.87	52.28	53.21	50.81	50.42	52.33	51.11	16.97	23.3	62.06	51.05
21	60.61	60.14	60.85	60.32	61.37	60.95	61.01	61.02	62.44	62.39	63.82	61.25	61.36	61.05	60.13	61.52	60.75	60.96	61.17	62		52.19	50.49	50.42	48.5	47.13	48.76	46.82	15.83	22.33	50.95	49.63
22	59.85	59.39	59.99	59.68	60.73	60.8	60.28	60.74	61.98	61.12	62.59	60.18	60.87	60.63	59.7	60.74	59.06	61.13	59.76	60.97	62		57.75	72.18	49.46	47.16	45.53	50.28	15.18	20.12	49.88	61.31
23	59.93	59.69	60.17	59.81	61.47	61.57	61.23	61.71	63.47	62.49	64.18	61.12	62.25	61.16	60.75	61.5	60.06	62.11	60.86	62.07	62.44	65.48		55.78	48.18	45.17	44.56	47.84	15.01	18.62	48.85	57.63
24	59.69	59.47	59.73	59.36	61.08	60.74	60.5	60.79	61.65	60.95	62.64	60.47	61.06	60.92	59.95	60.32	59.27	61.42	60.07	61.82	62.2	84.78	65.98		49.26	47.24	44.63	49.54	14.97	19.84	49.45	59.85
25	62.21	61.47	62.12	61.75	63.64	63.37	63.28	63.46	64.42	64.4	64.85	62.49	63.08	61.05	61.31	63	62.14	61.22	61.97	62.43	59.87	59.88	65.98	60.2		46.83	46.82	47.36	17.35	21.42	52.92	47.81
26	56.75	56.39	56.55	56.55	57.69	57.52	57.41	57.56	58.5	58.31	58.23	57.24	57.43	57.46	57.04	57.38	56.64	56.75	57.22	57.97	56.09	56.15	56.38	56.66	57.41		44.03	47.07	17.82	24.31	48.59	44.47
27	61.81	61.58	61.75	60.83	61.69	61.5	61.5	61.76	62.88	63.45	64.53	60.69	61.4	61.37	61.53	63.24	63.39	59.63	61.86	61.47	58.65	57.89	58.43	58.07	59.2	55.87		44.3	18.87	25.49	52.66	43.5
28	58.49	58.85	58.6	58.46	59.19	58.89	58.79	59.18	60.31	59.82	60.99	59.52	59.96	60.47	59.56	59.38	58.64	61.22	59.09	60.42	57.7	59.18	59.11	59.61	60.25	56.38	56.41		14.53	19.69	47.64	48.27
29	40.23	39.88	39.78	39.7	40.25	40.29	40.06	39.95	39.98	40.19	39.89	40.01	40.27	39.7	40.84	41.04	41.02	39.14	39.67	39.82	39.23	39.23	38.94	38.85	39.79	40.42	40.33	39.04		30.64	17.9	14.32
30	42.18	41.91	42.12	41.48	42.3	42.36	42.25	42.18	42.27	42.22	42.1	42.22	42.57	41.74	42.13	43.41	42.56	41.37	41.57	42.5	41.66	41.28	41.33	41.39	41.89	42.84	43.14	40.58	48.07		23.54	18.4
31	72.13	71.9	72.17	71.42	63.91	63.85	63.6	63.51	64.44	67.18	69.55	63.37	63.63	64.68	63.36	64.06	73.59	61.95	70.57	65.02	60.32	59.02	60.1	59.81	62.56	56.81	62.72	58.62	40.09	42.42		48.06
32	59.13	58.79	58.98	58.75	59.99	60.15	59.48	60.15	61.81	61.1	62.45	60.05	59.95	59.87	59.67	59.84	59.28	60.78	59.58	60.28	61.28	71.67	65.53	71.35	59.67	56.15	57.54	58.96	38.62	41.06	59.11	

Taxa; 1- *Rhodobacter capsulatus* ATCC 11166^T; 2- *Rhodobacter viridis* JA737^T; 3- *Rhodobacter maris* JA276^T; 4- *Rhodobacter aestuarii* JA296^T; 5- *Rhodobacter sphaeroides* 2.4.1^T; 6- *Rhodobacter johrii* JA192^T; 7- *Rhodobacter megalophilus* JA194^T; 8- *Rhodobacter azotoformans* KA25^T, 9- *Rhodobacter ovatus* JA234^T; 10- *Rhodobacter vinaykumarii* JA123^T; 11- *Rhodobacter veldkampii* ATCC 35703^T; 12- *Rhodobacter blasticus* DSM 213^T, 13- *Cereibacter changlensis* JA139^T; 14- *Defluviimonas denitrificans* DSM 18921^T; 15- *Falsirhodobacter* sp. alg1; 16- *Haematobacter missouriensis* CCUG 52307^T; 17- *Paenirhodobacter enshiensis* DW2-9^T; 18- *Pseudorhodobacter ferrugineus* DSM 5888^T; 19- *Thioclava pacifica* DSM 10166^T; 20- *Gemmobacter aquatilis* DSM 3857^T; 21- *Pararhodobacter aggregans* DSM 18938^T; 22- *Rhodobaca barguzinensis* alga-05^T; 23- *Roseicitreum antarcticum* CGMCC 1.8894^T; 24- *Roseinatronobacter thiooxidans* DSM 13087^T; 25- *Rhodovulum sulfidophilum* DSM 1374^T; 26- *Albimonas donghaensis* DSM 17890^T; 27- *Paracoccus denitrificans* DSM 413^T; 28- *Roseobacter litoralis* Och 149^T; 29- *Escherichia coli* ATCC 11775^T; 30- *Pseudomonas aeruginosa* DSM 50071^T; 31- *Sinorhodobacter ferrireducens* CCTCC AB2012026^T; 32- *Roseibaca ekhonensis* CECT 7235^T. *Rba. sphaeroides* clade member values in red color; *Rba. capsulatus* clade member values in light brown color; *Rba. veldkampii* clade (*Rba. veldkampii* and *Rba. vinaykumarii*) member values in pink color.
