# Supplementary material for: Taxogenomics Resolves Conflict in the Genus Rhodobacter: A Two and Half Decades Pending Thought to Reclassify the Genus Rhodobacter
Source: Front Microbiol. 2019 Oct 31;10:2480. doi: 10.3389/fmicb.2019.02480 (PMC6834548; doi:10.3389/fmicb.2019.02480)
Supplement: Supplementary file 2 [file Data_Sheet_2.pdf]

Taxogenomics Resolves Conflict in the Genus *Rhodobacter*: A Two and Half Decades Pending Thought to Reclassify the Genus *Rhodobacter*

[1#]Suresh G., [1#$]Tushar D Lodha., [1]Indu B., [2*]Sasikala Ch. and [1*]Ramana Ch.V.

[1]Department of Plant Sciences, School of Life Sciences, University of Hyderabad, P.O. Central University, Hyderabad-500 046, India.
[2]Bacterial Discovery Laboratory, Centre for Environment, Institute of Science and Technology, J. N. T. University Hyderabad, Kukatpally, Hyderabad-500085, India.
[$]Present address: National Centre for Microbial Resources, National Centre for Cell Sciences, Pune-411011, India.

#both the authors contributed equally

***Authors for correspondence: Ramana, Ch.V., Sasikala, Ch.**
E-mail: cvr449@gmail.com; sasi449@yahoo.ie

Running title: Reclassification of the genus *Rhodobacter*

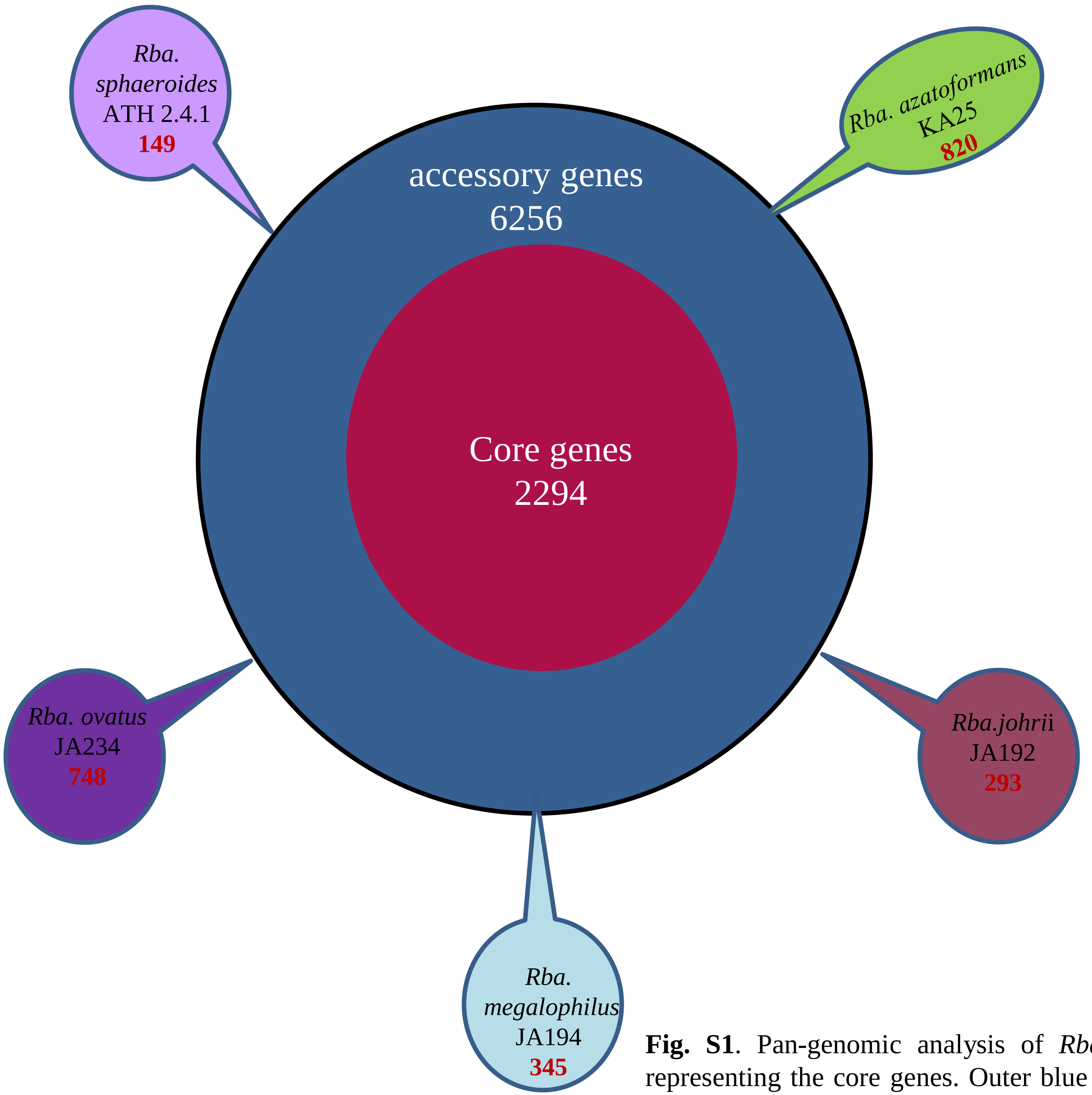

Fig. S1. Pan-genomic analysis of *Rba. sphaeroides* clade , Inner pink circle representing the core genes. Outer blue circle representing the accessory genes of all 5 organisms, and the buds representing the individual organisms unique genes

Pangenomic analysis of Clade II

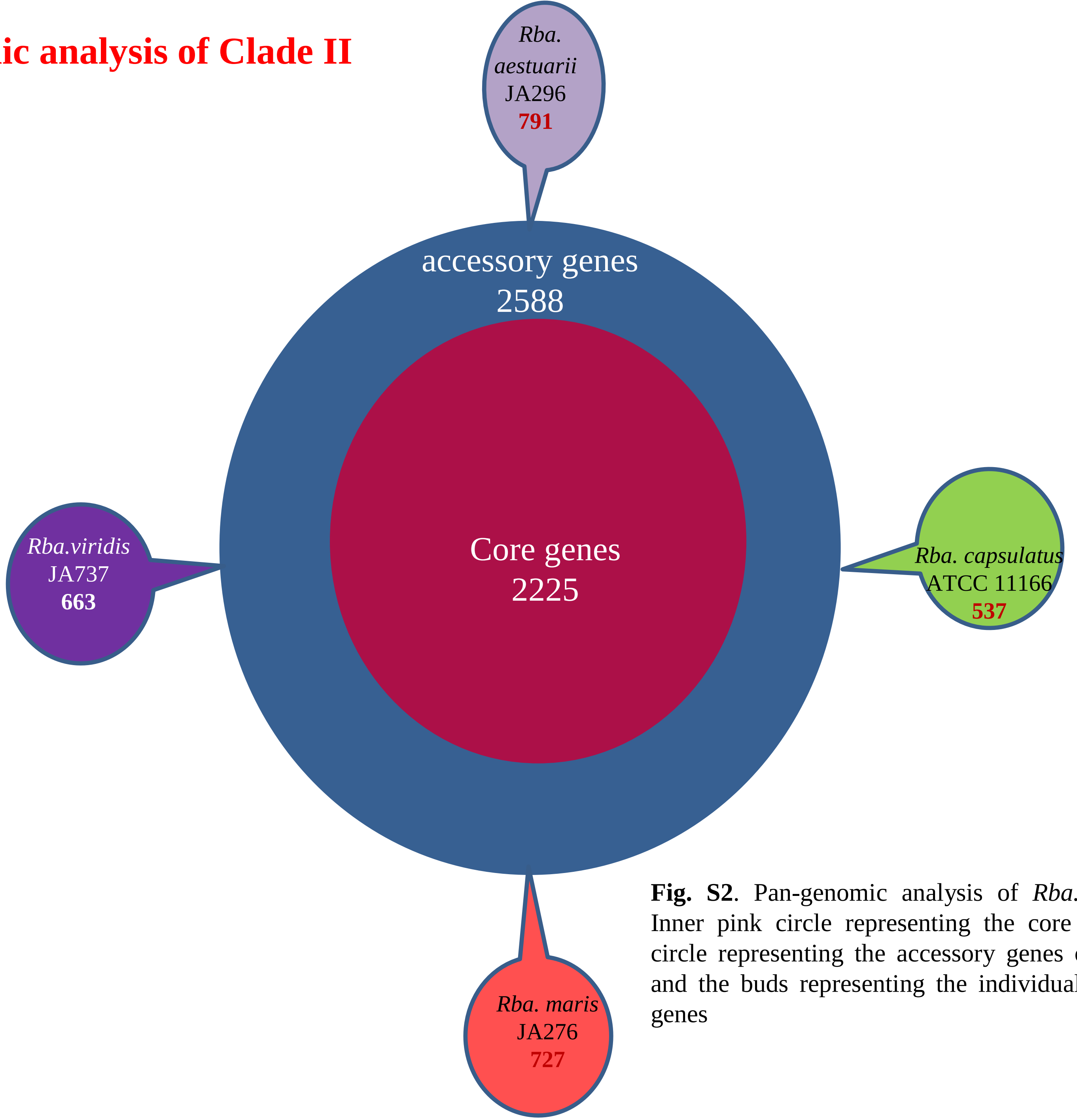

Fig. S2. Pan-genomic analysis of *Rba. capsulatus* clade, Inner pink circle representing the core genes. Outer blue circle representing the accessory genes of all 4 organisms, and the buds representing the individual organisms unique genes

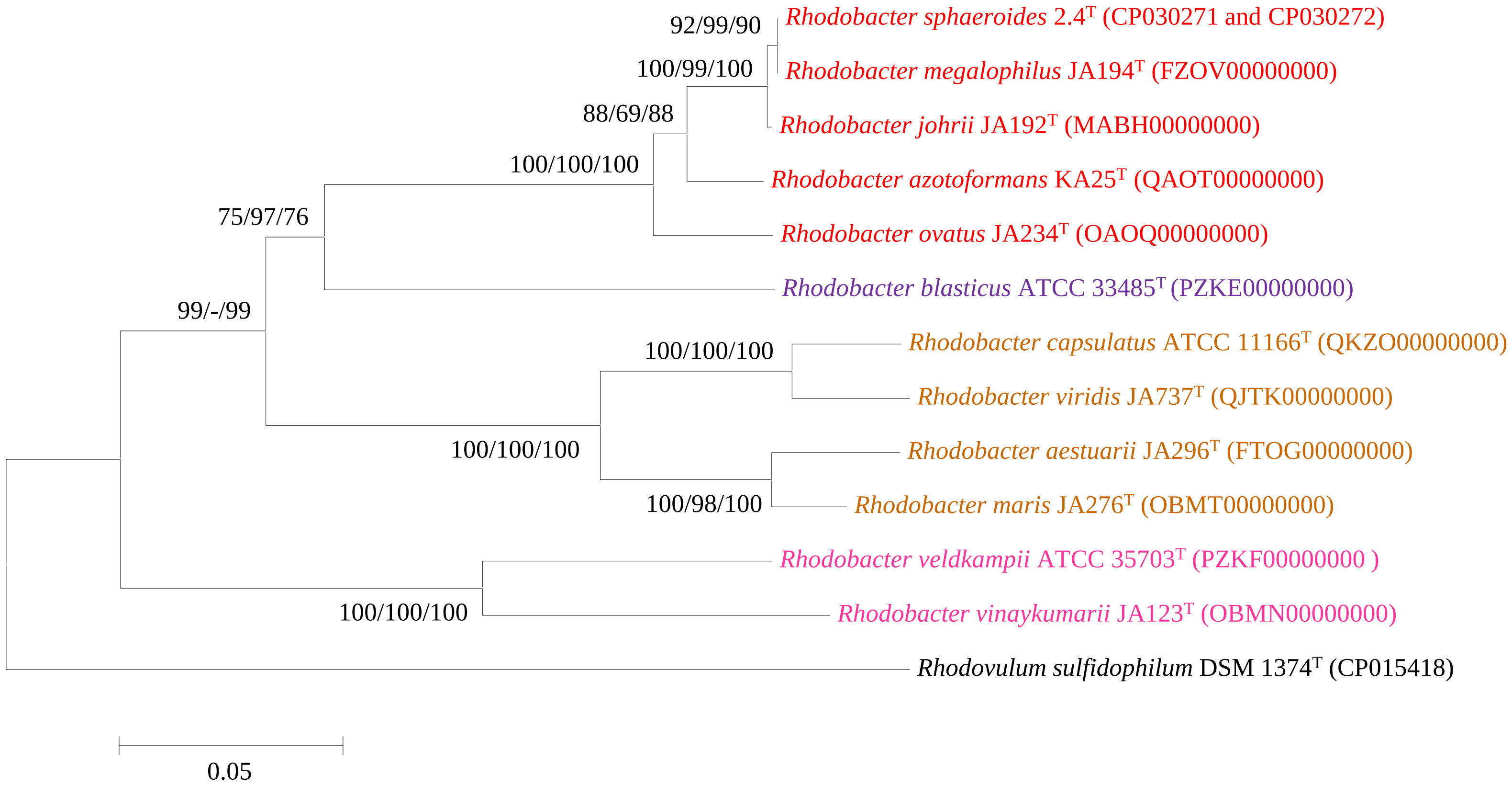

Fig. S3. Phylogenetic analysis Puf L and M concatenated protein sequences, tree was constructed using Mega 7 softawre. The evolutionary distances were computed using the poisson correction method. The bootsrap values are calculated by 1000 replicates and the values are represeting the bootstrap values of NJ/ML/ME tree.

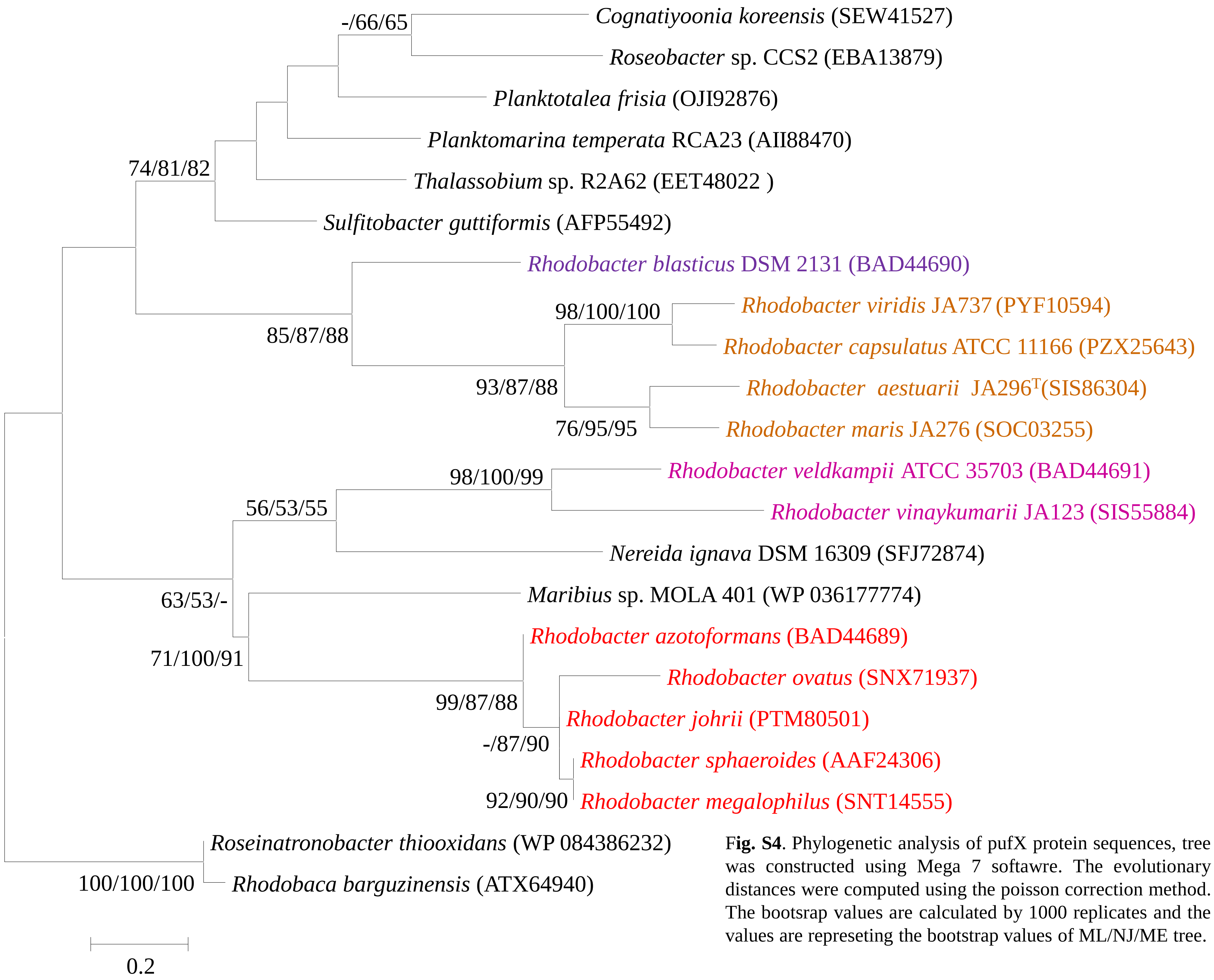

Fig. S4. Phylogenetic analysis of pufX protein sequences, tree was constructed using Mega 7 softawre. The evolutionary distances were computed using the poisson correction method. The bootsrap values are calculated by 1000 replicates and the values are represeting the bootstrap values of ML/NJ/ME tree.

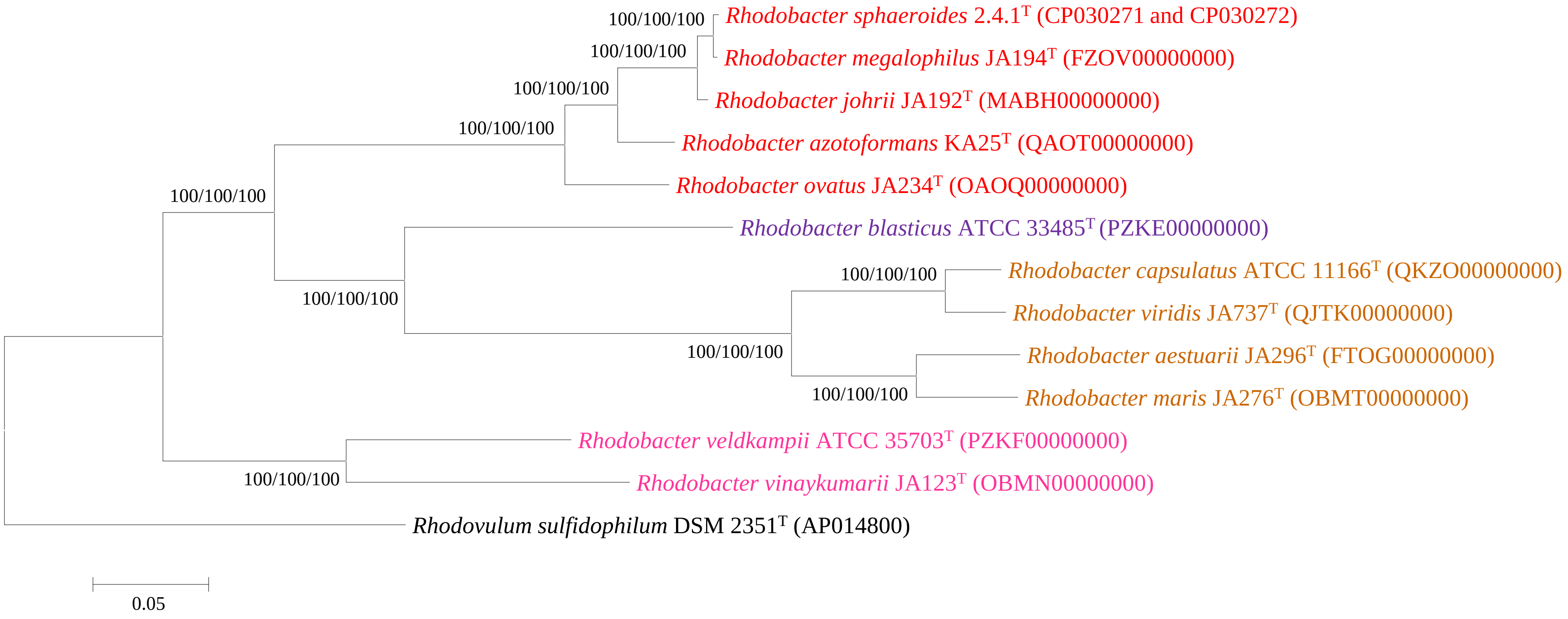

Fig. S5. PGC Phylogenetic analysis of PGC (Photosynthetic gene cluster) concatenated protein sequences, tree was constructed using Mega 7 softawre. The evolutionary distances were computed using the poisson correction method. The bootstrap values are calculated by 1000 replicates and the values are represeting the bootstrap values of NJ/ML/ME tree.

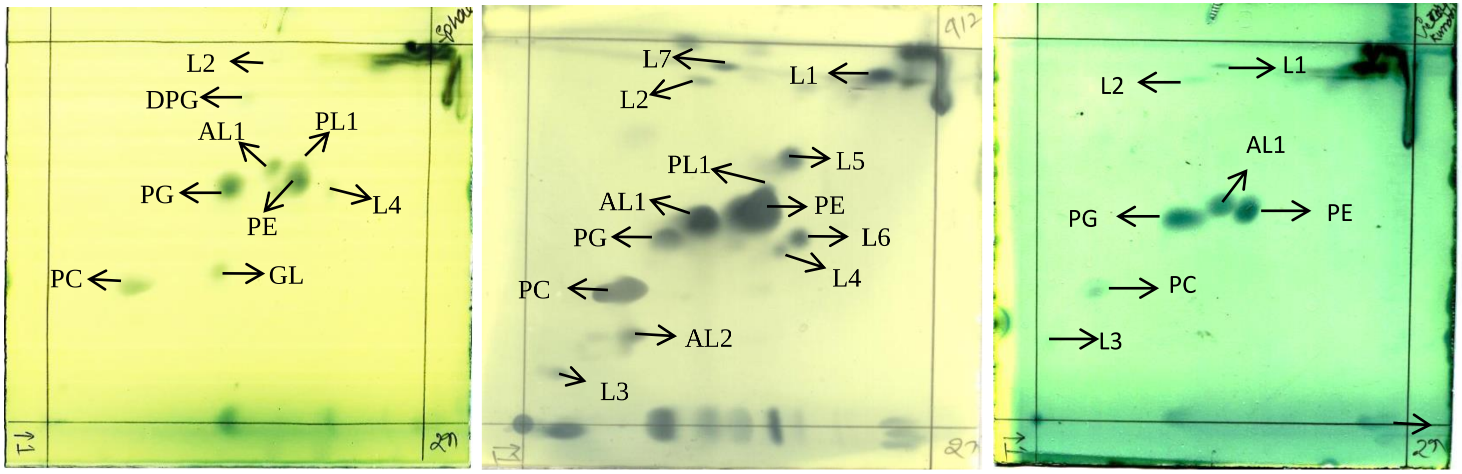

Clade I Clade II Clade III & Clade IV

Fig S6. Two-dimensional thin-layer chromatograms of whole cell polar lipids of clade I (*Rba. sphaeroides Rba. johrii, Rba. megalophilus*, *Rba. azotoformans*, *Rba. ovatus*, *Rba. alkalitolerans),* clade II (*Rba. capsulatus* , *Rba. viridis*, *Rba. azollae*, *Rba. sediminis*. *Rba. aestuarii*, *Rba. maris*, *Rba. lacus*) , Clade III (*Rba. blasticus*); Clade IV (*Rba. veldkampii* clade having *Rba. veldkampii* and *Rba. vinaykumarii*); PE, phosphatidylethanolamine; PG, phosphatidylglycerol; DPG, diphosphatidylglycerol; PC, phosphatidylcholine; PL1, uncharacterized phospholipid; AL1,2, uncharacterized aminolipids; GL, Glycolipid; L1-7 uncharacterized lipids.

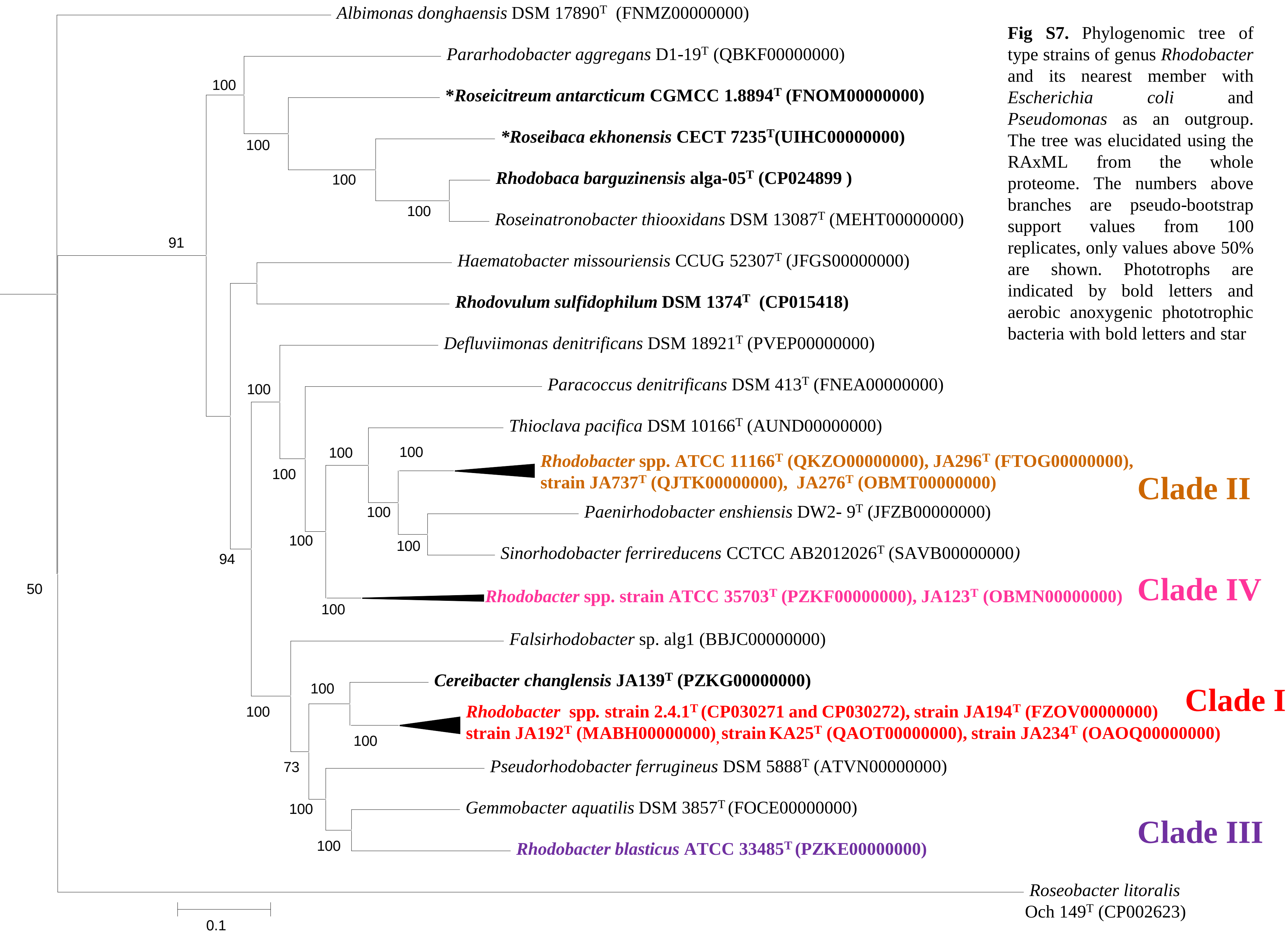

Fig S7. Phylogenomic tree of type strains of genus *Rhodobacter* and its nearest member with *Escherichia coli* and *Pseudomonas* as an outgroup. The tree was elucidated using the RAxML from the whole proteome. The numbers above branches are pseudo-bootstrap support values from 100 replicates, only values above 50% are shown. Phototrophs are indicated by bold letters and aerobic anoxygenic phototrophic bacteria with bold letters and star
